# A systematic review of adverse drug events associated with administration of common asthma medications in children

**DOI:** 10.1371/journal.pone.0182738

**Published:** 2017-08-09

**Authors:** James S. Leung, David W. Johnson, Arissa J. Sperou, Jennifer Crotts, Erik Saude, Lisa Hartling, Antonia Stang

**Affiliations:** 1 Division of Pediatric Emergency Medicine, Department of Pediatrics, McMaster University, Hamilton, Ontario, Canada; 2 Departments of Pediatrics, Emergency Medicine, and Physiology and Pharmacology, University of Calgary, Alberta Children’s Hospital Research Institute, Calgary, Alberta, Canada; 3 Cumming School of Medicine, University of Calgary, Calgary, Alberta, Canada; 4 Departments of Pediatrics, Emergency Medicine, Pediatric Emergency Research Institute, Calgary, Alberta, Canada; 5 Departments of Emergency Medicine and Pediatric Emergency Medicine, University of Calgary, Calgary, Alberta, Canada; 6 Alberta Research Center for Health Evidence, Department of Pediatrics, University of Alberta, Edmonton, Alberta, Canada; 7 Departments of Pediatrics, Emergency Medicine, and Community Health Sciences, University of Calgary, Alberta Children’s Hospital Research Institute, Calgary, Alberta, Canada; University of Nottingham, UNITED KINGDOM

## Abstract

**Objective:**

To systematically review the literature and determine frequencies of adverse drug events (ADE) associated with pediatric asthma medications.

**Methods:**

Following PRISMA guidelines, we systematically searched six bibliographic databases between January 1991 and January 2017. Study eligibility, data extraction and quality assessment were independently completed and verified by two reviewers. We included randomized control trials (RCT), case-control, cohort, or quasi-experimental studies where the primary objective was identifying ADE in children 1 month– 18 years old exposed to commercial asthma medications. The primary outcome was ADE frequency.

**Findings:**

Our search identified 14,540 citations. 46 studies were included: 24 RCT, 15 cohort, 4 RCT pooled analyses, 1 case-control, 1 open-label trial and 1 quasi-experimental study. Studies examined the following drug classes: inhaled corticosteroids (ICS) (n = 24), short-acting beta-agonists (n = 10), long-acting beta-agonists (LABA) (n = 3), ICS + LABA (n = 3), Leukotriene Receptor Antagonists (n = 3) and others (n = 3). 29 studies occurred in North America, and 29 were industry funded. We report a detailed index of 406 ADE descriptions and frequencies organized by drug class. The majority of data focuses on ICS, with 174 ADE affecting 13 organ systems including adrenal and growth suppression. We observed serious ADE, although they were rare, with frequency ranging between 0.9–6% per drug. There were no confirmed deaths, except for 13 potential deaths in a LABA study including combined adult and pediatric participants. We identified substantial methodological concerns, particularly with identifying ADE and determining severity. No studies utilized available standardized causality, severity or preventability assessments.

**Conclusion:**

The majority of studies focus on ICS, with adrenal and growth suppression described. Serious ADE are relatively uncommon, with no confirmed pediatric deaths. We identify substantial methodological concerns, highlighting need for standardization with future research examining pediatric asthma medication safety.

## Introduction

Wheeze is a common childhood problem, affecting one in three children before their third birthday, and almost 50% by 6 years of age.[1, 2] To treat this wheeze, asthma medications are frequently prescribed to children, regardless of a clear-cut diagnosis of asthma, which is particularly difficult in pre-school aged children and infants who present with bronchiolitis.[3] Considering their frequent labeled and off-labelled use, an understanding of adverse drug events associated with asthma medications is crucial to safe medical practice.

Defined by the World Health Organization (WHO) as “any untoward medical occurrence that may present itself during treatment with a medicine but which does not necessarily have a casual relationship with the treatment”, Adverse Drug Events (ADE), are a measure of harm from medication administration.[4, 5] These ADE include harm from appropriately administered medications at appropriate doses (Adverse Drug Reactions, ADR), along with harm from inappropriately administered medications (Harmful Medical Error)[4].

The Global Initiative for Asthma (GINA) describes the following classes of medications to be used with asthmatic patients: short-acting beta agonists (SABA), inhaled corticosteroids (ICS), long-acting beta agonists (LABA), leukotriene receptor antagonists (LTRA), systemic corticosteroids (SCS) and IgE Immunomodulators (Anti-IgE).[6] Despite their common use,[7, 8] there is a paucity in understanding ADE associated with common asthma medications in children. For example, a broad systematic review focusing on ADR in children specifically excluded studies focusing on asthma.[9] We conducted a systematic review with the primary objective to determine the frequency of all ADE associated with commonly used asthma medications in children. Our secondary objectives were to describe the causality, severity and preventability of these ADE.

## Methods

We designed and conducted our systematic review following guidelines published by the Preferred Reporting Items for Systematic Reviews and Meta-analyses (PRIMSA) consortium.[10]

### Literature search

A medical research librarian (AM), in association with medical experts from the research team (AS, JL, DJ), developed the primary search strategy. We completed a systematic search of the literature from January 1991 to January 2013, built on three concepts: “asthma” OR “adverse drug events” AND/OR “asthma medications” (**S1 Protocol)**. 1991 was selected as the start of our search period due to the publication of the landmark Harvard Medical Practice II review, which highlighted iatrogenic harm from medications and led to increasing research on ADE and patient safety. A start date of 1991 was also selected to ensure asthma medications reflected current care. Six databases were searched: Medline, Central, EMBASE, PubMed, Web of Knowledge, and International Pharmaceutical Abstracts. We also searched online human clinical trial registries from U.S. National Institutes of Health, National Institute for Health Research and the WHO. Prior to publication, we completed an updated search of the literature from November 2012 to January 2017, with MeSH terms updated to reflect narrower subheadings added since 2012 **(S2 Protocol)** (RF). Two databases where searched: Medline and EMBASE.

### Study selection

After removal of duplicate studies, two independent reviewers (JL, CS) screened titles and abstracts. Any citation which either reviewer thought should be included, or unclear for inclusion was identified for full text screening. Subsequently, two reviewers (JL, CS or AS) independently reviewed full texts of potentially eligible articles for final inclusion and data extraction. Disagreements on studies to include were resolved by two-thirds consensus between the three reviewers.

At both selection stages, reviewers followed a screening protocol with pre-defined eligibility criteria (**S3 Protocol**) including: primary study objective, study design, and asthma medication studied. As quality of identification, assessment and reporting of ADE may be less rigorous in studies where ADE were studied as a secondary objective we decided, a priori, that included studies needed to specify identifying ADE as the primary objective. Similarly, to maximize data quality, we included randomized control trials (RCT), case-control, cohort, quasi-experimental study designs and excluded case reports, case series, cross-sectional studies and phase III clinical trials. We also excluded studies that: 1) did not provide data on the frequency of ADE; 2) only presented aggregated “pediatric and adult” data, without separate pediatric subgroup analysis; 3) included only neonates (less than 1 month of age) because of their pharmacodynamic and pharmacokinetic differences from older children; 4) provided data only on experimental medications; and 5) reported only on theophylline. Due to funding and resource issues, we only reviewed articles in English and were unable to perform a manual search of bibliographic references from retrieved papers. Of note, phase III clinical trials were excluded as a primary objective of these studies includes confirming treatment efficacy, rather than identifying ADE, and drugs are experimental at the time of study publication.

The above process was repeated during our rescreening process, with three reviewers (JL, ES, JC) independently screening newly searched titles and abstracts, followed by a full text screen for study inclusion and data extraction.

### Data extraction

Standard data abstraction forms were used for each included study. Four independent reviewers (JL, AJS, CS or MS) conducted initial data extraction; each initial review was verified independently by a second reviewer (JL or AJS). Extracted data included: publication information, funding sources, study design, study group demographics including study setting, age range, sample size, and medication exposure duration. We also extracted our primary and secondary outcome data: medications studied, adverse drug event type and frequency (primary outcome), organ system involved, medication error analysis, and adverse drug reaction analysis. The same 2-step protocol with data extraction and independent verification was repeated for articles identified during rescreening (JL, ES, JC).

### Quality assessments

The primary data abstractor (JL, AJS, CS or MS) completed methodological quality assessments using the Newcastle-Ottawa Quality Assessment Scale[11] for case-control and cohort studies, and the Cochrane Risk of Bias tool for RCT.[12] We did not complete a quality assessment for included abstracts due to lack of presented information. The primary abstractor also assessed quality of ADE content using a previously published tool, referred to as the Smyth Adapted ADE scale. This tool assessed a study’s methods for identifying ADE causality, severity and preventability.[9] A second reviewer (JL, AJS) independently verified all quality assessments for accuracy. Quality assessments were also carried out during rescreening (JL, ES, JC).

### Analysis

We conducted a qualitative and limited quantitative analysis on extracted data. Study characteristics, including study design, study setting, drug exposure, population size, inclusion of a control group, and quality assessments are presented as medians, counts and proportions as appropriate. The primary outcome, ADE frequency, is presented as a proportion calculated from total number of exposed patients within the study. All described ADE were categorized by drug class and by primary organ system affected. For the secondary outcomes we presented the number of cases of severe ADE, as well as the proportion of studies that included a standardized method for assigning degree of ADE causality, rating severity with a standardized severity scale and determining ADE preventability using standardized methods. We also highlighted ADE frequencies of particular clinical concern and controversy based on prior literature, such as adrenal and growth suppression in ICS, deaths associated with LABA and neuropsychiatric ADE in LTRA. We only used data from the groups exposed to the drug and did not provide comparative/relative measures between drugs, when available, as this was beyond the primary objective of our study, not all studies provided control/placebo data and several studies compared outcomes of medications in patients exposed to different drug classes (i.e. compared ADE between an ICS and combined LABA/ICS).

## Results

### Study selection

**Fig 1:**
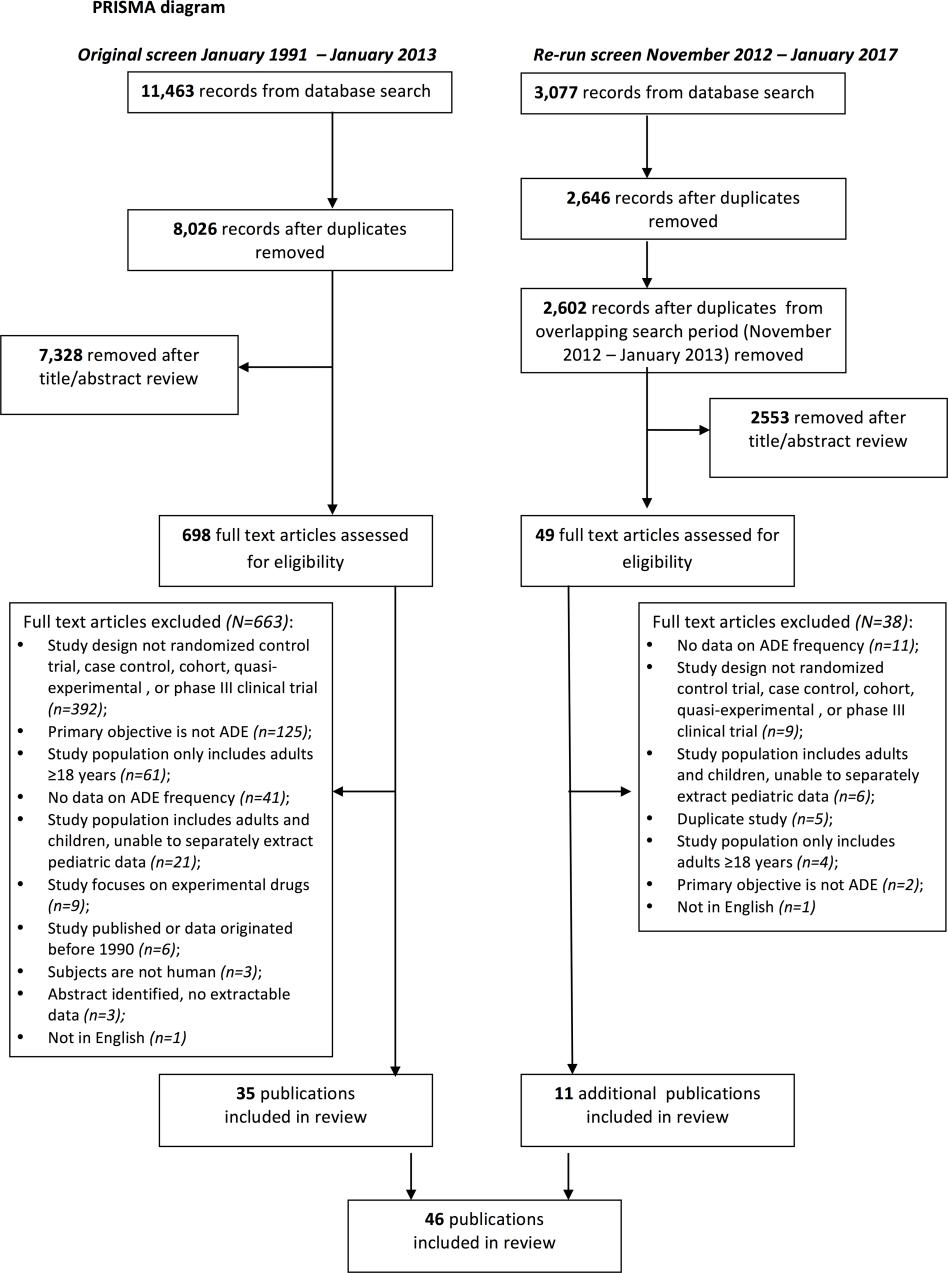
PRISMA flow diagram for included studies.

Our original database search generated 11,463 results, of which 3,437 duplicates were removed (**Fig 1**). The titles and abstracts of 8,026 studies were screened, with 7,328 studies excluded. The full texts of 698 articles were reviewed, with 35 articles [13–47] meeting all inclusion criteria. Our repeated database search generated 3077 results, with 431 duplicates removed and an additional 44 articles removed as they were duplicates from the overlapping search period from November 2012 to January 2013. The full texts of 49 articles were reviewed, with an additional 11 articles meeting inclusion criteria [48–58] increasing the total number of included articles to 46 (**Fig 1**).

All studies were from published, peer-reviewed sources. Three published abstracts were included. No additional studies were identified from online human clinical trial registries.

### Study characteristics

Amongst the 46 included studies (**Table 1**), six types of study design were noted: 24 randomized control trials (17–19, 21, 23–26, 28–30, 33, 36–43, 45–47,[57], 15 (33%) cohort studies [13–15, 20, 22, 27, 48, 49, 51–56, 58], 4 pooled analyses of open-label RCT [31, 32, 35, 44], 1 case-control study[50], 1 open-label trial [16] and 1 quasi-experimental study [33]. The authors of 29 studies declared pharmaceutical industry funding; 29 studies were conducted in North America and 33 studies in a clinic setting.

**Table 1:** Summary of included studies.

Study (Reference)	Original search result	Funding	Drug (months of medication exposure)	Study Design	Study setting; Country	Sample size; age range	Control data	Primary objective
Abusamra 2013 [48]	No	NR	Salbutamol (inhaled, nebulized or IV) (<1day)	Abstract; Retrospective cohort	ICU; United Kingdom	36; NR	No	Identify incidence of lactic acidosis following salbutamol therapy and relation to patient characteristics and dose
Baumann 2014 [49]	No	NR	Inhaled corticosteroid NOS (6–10)	Abstract; cohort	NR; Serbia	150; 7-18y	Yes	Determine growth and nutritional status of male children with asthma on long term therapy of ICS
Behbehani 2005 [13]	Yes	NR	Beclomethasone propionate and/or budesonide (6)	Prospective cohort	Clinic; Kuwait	95; 1.25-12y	No	Incidence of posterior subcapsular cataract and ocular hypertension in children on ICS
Bentur 2000 [14]	Yes	NR	Beclomethasone propionate or budesonide (2)	Prospective cohort	Clinic; Israel	55; 6-36mo	Yes	Effect of ICS on the urinary calcium excretion rate in young asthmatic children
Bentur 2003 [15]	Yes	NR	Budesonide (2)	Prospective cohort	Clinic; Israel	25; 3-6y	No	To determine if ICS induces hypercalciuria in children with asthma
Berger 2003 [16]	Yes	Industry	Omalizumab (12)	Prospective open-label trial	Clinic; USA	225; 6-12y	Yes	Overall safety and tolerability of omalizumab in asthmatic children
Berger 2005 [17]	Yes	Industry	Budesonide [45]	Prospective RCT	Clinic; USA	141; 6-12mo	Yes	Compare safety of budesonide to placebo in infants with moderate persistent asthma or recurrent wheeze
Berger 2010 [18]	Yes	Industry	Formoterol + Budesonide or budesonide (6.5)	Prospective open-label RCT	Clinic; USA	158; 6-12y	No	Compare safety of budesonide+formoterol pMDI to budesonide children 6–11 years with persistent asthma
Bisgaard 2004 [19]	Yes	Industry	Fluticasone proprionate or sodium cromoglycate (12)	Prospective open-label RCT	Clinic; Denmark	625; 12-47mo	No	Assess the 1-year safety and efficacy in children 1–3 years with mild to moderate recurrent wheeze
Cavkaytar 2015 [50]	No	NR	Fluticasone propionate, budesonide or ciclesonide (>1.5)	Retrospective Case Control	Clinic; Turkey	91; 0-18y	No	Determine rate of adrenal suppression in children taking moderate to high doses daily ICS
Chiang 2000 [20]	Yes	NR	IV Terbutaline (NR)	Prospective cohort	ED or ICU; USA	29; 2.6–15.6y	No	Examine cardiac toxicity associated with IV terbutaline in children with severe asthma
de Benedictis 2001 [21]	Yes	Industry	Fluticasone propionate or beclomethasone propionate (12)	Prospective RCT	Clinic; Italy	343; 4-11y	No	Compare long-term effects of ICS treatment on growth in asthmatic children
Dubus 2001 [22]	Yes	NR	Beclomethasone propionate or budesonide (15)	Prospective cohort	Clinic; France	639; 3mo-16y	No	Compare local side effects from ICS
Egeland 2013 [51]	No	NR	Magnesium sulphate (<1 day)	Prospective cohort	ICU; USA	57; 2-18y	Yes	To assess the safety and pharmacokinetics of high-dose MgSO4 infusion in pediatric patients with status asthmaticus.
Erdem 2015 [52]	No	NR	Leukotriene antagonist, type not specified (NR)	Retrospective cohort	Clinic; Turkey	1024; age range not stated, mean 6.5y	No	Evaluate side effects of leukotriene antagonists
Fagbuyi 2016 [53]	No	NR	Continuous albuterol nebulization (8.2±4.9 hours)	Prospective cohort	ED/ICU; USA	50; 3.8–12.6y	No	Determine occurrence of diastolic hypotension and cardiac changes in children with mod/severe asthma needing continuous nebulization
Ferguson 2007 [23]	Yes	Industry	Fluticasone propionate or budesonide (12)	Prospective RCT	Clinic; Canada	233; 6-9y	No	Compare effect of equivalent doses ICS in pre-pubescent, asthmatic children
Hinkle 2011 [24]	Yes	Industry	Arformoterol or levalbuterol (1 day)	Prospective RCT with open-label crossover	Clinic; USA	53; 2-11y	No	Determine safety and tolerability in asymptomatic patients with stable asthma
Kaashmiri 2010 [25]	Yes	Industry	Albuterol hydrofluroalkane (1 day)	Prospective RCT	ED; USA	87; 0-24m	No	Assess medication safety in children <2 years old with acute wheeze and obstructive airway disease
Kearns 2008 [26]	Yes	Industry	Montelukast (0.5)	Prospective RCT	Clinic; USA	12; 1-3m	Yes	Determine safety of montelukcast in children compared to placebo
Kelly 2008 [27]	Yes	Government + Industry	Oral corticosteroids or ICS (84)	Prospective cohort	NR; USA	877; 5-12y	Yes	Assess effects of bursts of oral corticosteroids and long-term use of inhaled corticosteroids on bone mineral accretion
Kenyon 2014 [54]	No	Government	Albuterol continuous neb (1 day)	Retrospective Cohort	Inpatient; USA	3137; 2-17y	Yes	Assess clinical deterioration and adverse medication effects associated with continuous nebulized albuterol in an inpatient, non-ICU/ED setting
Kerwin 2006 [28]	Yes	Industry	Albuterol (1)	Prospective RCT	Clinic; USA	77; 24–47 mo	Yes	Assess safety of albuterol at two different doses
Kim 2006 [29]	Yes	NR	Orapred or generic oral prednisolone (1 day)	Prospective RCT	ED; USA	188; 2-10y	No	Compare incidence of vomiting
Kuusela 2000 [30]	Yes	Industry	Terbutaline sulphate or bambuterol hydrochloride [45]	Prospective RCT	Clinic; Finland	155; 2-6y	No	Compare safety at equivalent doses of medications for three months
Leflein 2001 [31]	Yes	Industry	Budesonide (12)	Pooled analysis of 3 open-label trails	Clinic; USA	670; 8m-9y	Yes	Determine long-term safety of lowest individual maintenance dose
Leflein 2005 [32]	Yes	Industry	Budesonide (21)	Pooled analysis of 3 open-label trails	Clinic; USA	198; 2-10y	Yes	Assess long term medication safety in children with persistent asthma
MacKenzie 1994 [33]	Yes	Industry	Fluticasone propionate (12)	Quasi experimental	Clinic; UK	257; 4-17y	No	Long-term tolerability in managing childhood asthma
Malone 2005 [34]	Yes	Industry	Fluticasone+salumetrol or fluticasone [45]	Prospective RCT	Clinic; USA	203; 4-11y	No	Compare safety of medications
Milgrom 2011 [35]	Yes	Industry	Omalizumab (NR)	Pooled analysis of two RCT	Not stated; USA	926; 6-12y	Yes	Evaluate safety profile in children with moderate-severe persistent asthma
Noonan 2009 [36]	Yes	Industry	Mometasone furoate or beclomethasone propionate (12)	Prospective open-label RCT	Clinic; USA`	233; 4-11y	No	Compare long-term safety of medications in children with mild-to-moderate asthma
Pauwels 2003 [37]	Yes	Industry	Formetorol or salbutamol (6)	Prospective open-label RCT	Clinic; Belgium	3290; 6-17y	No	Assess safety of medications
Perry 2014 [55]	No	NR	Mometasone + formorterol (6)	Abstract; cohort	Clinic, Canada	101; 3.2–17.6	No	Identify prevalence of adrenal insufficiency in children treated with Zenhale (mometasone-formoterol)
Roux 2003 [38]	Yes	Industry	Fluticasone priopionate or Nedocromil sodium (24)	Prospective open-label RCT	Clinic; France	15; 6-14y	No	Assess safety of medications on bone health
Sarniak 2013 [56]	No	NR	Albuterol (continuous nebulization) (1 day)	Retrospective cohort	Transport/ ICU; USA	154 (90 transport, 64 PICU); 2-18y	No	Determine prevalaence of diastolic hypotension in patients with high dose inhaled albuterol in transport and ICU
Silverman 2006 [39]	Yes	Industry	Budesonide (36)	Prospective RCT	Clinic; UK	1981; 4-10y	Yes	Describe the safety and tolerability in low-dose inhaled budesonide
Skoner 2005 [40]	Yes	Industry	Levalbuterol or racemic albuterol (1)	Prospective RCT	Clinic; USA	211; 2-5y	Yes	Compare safety of medications in children 2–5 years with asthma
Skoner 2008 [41]	Yes	Industry	Ciclesonide (12)	Prospective RCT	NR; USA	661; 5.5–9.1y	Yes	Assess effects of ciclesonide on growth in children with asthma.
Skoner 2010 [42]	Yes	Industry	Mometasone furoate (1)	Prospective RCT	Clinic; USA	50; 6-11y	Yes	Evaluate effects of higher doses of mometasone furoate on HPA axis in children with mild asthma
Stempel 2016 [57]	No	Industry	Fluticasone propionate+ salmetrol or Fluticasone propionate (2)	Prospective RCT	Clinic; USA	6208; 4-11y	Yes	Perform a large safety trial determining of fluticasone propionate+salmeterol is noninferior to fluticasone for risk of serious asthma ADE
van Adelsberg 2005 [43]	Yes	Industry	Montelukast (1.5)	Prospective RCT	Clinic; USA	256; 6-24m	Yes	Evaluate the safety and tolerability of monteleukast oral granules compared to placebo
Watson 1994 [44]	Yes	NR	Ipratropium bromide or albuterol (1 day)	Pooled analysis of 2 studies: RCT and open-label cohort	Clinic (RCT), Hospital (Cohort); Canada	46; 6-17y	No	Determine effects of medications on intraocular pressure in children with outpatient managed asthma, and admitted to hospital for acute asthma exacerbations
Weinstein 1997 [45]	Yes	Industry	Salmeterol (1 week)	Prospective RCT	Clinic; USA	243; 4-11y	Yes	Safety and efficacy of medications after treatment
Wisecup 2015 [58]	No	NR	Albuterol continuous nebulization (<1)	Retrospective cohort	Inpatient, ICU; USA	166; 2-11y	No	Determine prevalence of diastolic hypotension in patients with continuous albuterol nebulization
Wolthers 2001 [46]	Yes	Industry	Beclomethasone propionate (2.5)	Prospective open-label RCT with 2-period crossover	Clinic; Denmark	63; 5-11y	No	Assess short-term lower leg growth in children with asthma treated with belcomethasone propionate
Zarkovic 2000 [47]	Yes	Industry	Terbutaline sulphate and/or bambuterol hydrochloride (12)	Open-label RCT	Clinic; Austria	141; 1-13y	No	To evaluate safety of medications

NOS = not otherwise specificed; NR = Not Reported; y = years; m = months; ICS = inhaled corticosteroids; RCT = Randomized Control Trial; ED = Emergency Department; ICU = Intensive Care Unit; HPA = Hypothalmic-Pituitary-Adrenal

24 studies examined ICS, 10 SABA, 3 LABA, 3 combined ICS + LABA, 3 Leukotriene Receptor Antagonists 2 oral beta agonists, 2 IV cromoglycates, 2 subcutaneous anti-IgE, 2 systemic corticosteroids, 1 IV Magnesium Sulphate, and 1 anticholinergic asthma medications. Drug exposure durations ranged from less than 1 day [51, 53, 54, 56, 58, 59] to 84 months [27], and 11 studies report drug exposure durations greater than or equal to 12 months [16, 19, 21–23, 31, 32, 36, 38, 39, 47]. Study population sizes ranged from 12 [26] to 6208 [57] participants with a median of 162 participants. Only 14 studies report data on ADE from a control group [14, 16, 17, 27, 28, 31, 32, 35, 39–43, 45], with 7 of these studies focusing on inhaled corticosteroids.

### Primary outcome

We were unable to complete a meta-analysis due to the heterogeneity of study designs and results. A detailed index of ADE descriptions and frequencies organized by medication class and name is provided review in **S1 Table.** Of note, this index reports sample size (i.e. denominator), n, rather than number of cases reported (i.e. numerator) as an indication of study power. We distinguished ADE with 0% frequency from “not reported (NR)”, with 0% indicating that an ADE was monitored for but not observed and NR indicating that the ADE was not monitored and not observed.

A summary table of medications included in our systematic review, dosage range in reported studies, number of ADE and organ systems involved, organized by asthma drug class is provided (**Table 2).**

**Table 2:** Summary of medications included in study and described ADE.

Medication Name	Dosage Range	Drug Class	Number included studies	Total ADE	Number of ADE description by organ system affected												
					GI	Resp	CVS	Derm	CNS	Renal	Psych	MSK	ENT	Heme	Ophth	Endo	Other
Budesonide	150–2000 mcg/day	ICS	9	55	7*	7*	0	2	2	6*	1	2*	10*	3*	2	4	9*
Fluticasone propionate	200–400 mcg/day	ICS	8	56	5*	7*	1	4*	3*	1*	1	4*	11*	1	1	10	7*
Mometasone furoate	200–800 mcg/day	ICS	2	28	2	3	1	2	3	1		1	9		1		5*
Beclomethasone propionate	168–1008.3 mcg/day	ICS	3	24		6			1				9			4	4*
Ciclesonide	40–160 mcg/day	ICS	2	4									1	1		1	1
ICS NOS		ICS	4	7						1					1	5	
Prednisolone sodium phosphate	2mg/kg (max 60mg)	Systemic steroid	1	1	1												
Prednisolone	2mg/kg (max 60mg)	Systemic steroid	1	1	1												
Oral corticosteroid NOS	1 to >5 courses	Systemic steroid	1	1												1	
Salbutamol	90mcg TID– 360mcg/dose x 6 doses over 3 hours	SABA -intermittent	3	23	3	5*	6		2	1			2				4*
Levalbuterol	310mcg TID– 630 mcg/dose x 3 doses over 1 hour	SABA—intermittent	2	9	1	1			2		1*	1					3
Racemic Albuterol	1.25mg (if <33 pounds), 2.5 mg (if >33 pounds) TID	SABA—intermittent	1	2		1*			1		0						
Terbutaline IV	10 mg/kg bolus, 0.4mg/kg/min	SABA—continuous	1	4					4								
Salbutamol (continuous nebulization)	0.34–0.50 mg/kg/hr	SABA–continuous	5	12		1	8			2							1
Terbutaline sulphate	0.225 mg/kg/day to 7.5 mg /day maximum	PO B-agonist	2	7	1	1			1	1	1		1		1	0	1
Bambuterol	10–20 mg/day	PO B-agonist	2	9	1	2			2	0	1		1		0	1	1
Arformeterol	7.5–15 mcg/dose x 3 doses	LABA	1	9	2	1			1			1	1				3
Formeterol	4.5 mcg/dose PRN	LABA	1	2			1										1*
Salmeterol xinafolate	21–42 mcg/dose BID	LABA	1	3		1			1								1
Budesonide + Formeterol	Budesonide (320 mcg/day) + Formeterol (9 mcg/day)	LABA+ICS	1	10	2	3*	3			0	1		1	0			
Fluticasone propionate + Salmeterol	Fluticasone propionate (200 mcg/day) + Salmeterol (100 mcg/day)	LABA+ICS	1	24	3	3	1		1	1	1	1	7	1		1	4*
Mometasone + Formeterol	200-800mcg/day	LABA+ICS	1	1												1	
Ipratropium bromide + Saline (neb)	(250–500 mcg/dose) + Saline	Anticholinergic	1	5			1		1				2		1		
Ipratropium bromide + Albuterol (neb)	(250–500 mcg/dose) + (0.1 mg/kg)	Anticholinergic	1	5			1		1				2		1		
Montelukast	4–8 mg/day	LRTA	2	12	3	2	1						1		1		4*
LTRA NOS		LRTA	1	13	1			2	3		6						1
Sodium cromoglycate	20 mg/day	Cromoglycate	1	5		1							0		0	3	1*
Nedocromil sodium	8mg/day	Cromoglycate	1	9	1	3		0				0	3				2*
Omalizumab	75 mg q2weeks–375mg q2weeks	Anti-IgE	2	45	7*	4*		4*	1	0		7*	9*	4	1		8*
Magnesium sulphate IV infusion	50-70mg/kg bolus followed by 40mg/kg/h infusion x4h	Other	1	4	1			1									2
CFC propellant + beclomethasone		Other	1	7		1		1	1				2		0		2
HFA134a propellant + beclomethasone		Other	1	9		2		0	1				2		2		2

Resp, Respiratory; GI, Gastrointestinal; CVS, Cardiovascular; Derm, Dermatology; CNS, Central Nervous System; Psych, Psychiatric; MSK, Musculoskeletal; ENT, Ear, Nose and Throat; Heme, Hematologic; Opth, Ophthalmologic; Endo, Endocrine; ICS, Inhaled Corticosteroids; SABA, Short Acting Beta-Agonist; LABA, Long Acting Beta-Agonist; PO B-agonist, Oral Beta-Agonist; NOS, Not Otherwise Specified; LRTA, Leukotrience Receptor Antagonist; IV, Intravenous; Neb, Nebuliation; CFC:,Chloroflurocarbon; HFA, Hydrofluroalkane

* denotes a serious ADE was encountered in this organ system

The five most frequently studied medication classes are: SABA, LABA, ICS+LABA, LTRA and ICS.

Four SABA are studied in two routes of administration: intermittent or continuous administration. Combined, 12 studies with SABA data report 50 ADE that affect nine organ systems. Three studies on report an overall ADE frequency associated with intermittent SABA, ranging between 34.6–52% with salbutamol/albutarol [28] and 0–61% with levalbuterol[24, 40], with only 6% of these ADE thought to be drug-related [24](**S1 Table**). No overall ADE frequency or drug-related ADE frequency is reported with continuously administered SABA. The three most common ADE associated with intermittent SABA are anxiety (range 0–52%, levalbuterol), tachycardia (range 13.6–14%, salbutamol), and supraventricular ectopy (range 0–14%, salbutamol) (**S1 Table**). With continuous SABA, 50% of ADE affected the cardiovascular system, with the three most common ADE being tachycardia (range 94–95%, salbutamol), diastolic hypotension (range 66–98%, salbutamol), and lactic acidosis (80.6%, salbutamol).

Fourteen ADE affecting seven organ systems were reported with three LABA medications in three studies. One study reported overall ADE associated with the LABA, ranging from 14–20% with arformeterol, with only 4–5% judged to be drug related.[24] The three most common ADE are nonspecific lab abnormalities (range 9–10%, salmeterol), asthma exacerbations (range 7–9%, salmeterol), and nonspecific infection (range 2–8%, arformeterol) (**S1 Table**).

With ICS + LABA, 35 ADE affecting 11 organ systems are reported to be associated with three medications. Only one study [34], on fluticasone propionate + salmeterol, reported an overall ADE proportion of 59%, with no reported data on the frequency of ADE thought to be drug related. The three most common ADE are headache (20%, fluticasone + salmeterol), adrenal insufficiency (14.9%, mometasone + formoterol), upper respiratory tract infection (range 1–10%, fluticasone + salmeterol) (**S1 Table**).

Leukotriene antagonists (LTRA) are associated with 25 ADE affecting nine organ systems in three studies. Thirty-six percent of these ADE relate to the neuro-psychiatric system, including headache (<1%), hyperkinesis (<1%), seizure (<1%), appetite changes (<1%), anxiety/nervousness (<1%), fatigue (<1%), hallucinations (<1%), sleep disorder (<1%) and nyctophobia (<1%) [52]. One study commented on overall frequencies of ADE at 4%, but stated no frequency of drug-related ADE [52]. The most common ADE are upper respiratory tract infection (range 0–55%, montelukast), abnormal liver enzymes (range 14.3–40%, montelukast). (**S1 Table**)

Finally, 174 ADE affecting 13 organ systems are described for the five studied ICS (Table 2). The majority of ADE data in our review relates to ICS 174/406 (43%) of ADE associated with the drug class. Authors report an overall proportion of any ADE associated with ICS was reported in five studies ranging between 83.8–98% with budesonide, 15.7–57% with fluticasone and 90–95% with ciclesonide. [23, 31, 32, 41]. Frequency of ADE thought to be drug-related were reported only with fluticasone propionate in two studies, ranging from 4–23%. [19, 38]. As the majority of data focuses on ICS, we selected and highlight outcomes of particular clinical significance from our ADE Index (S1 Table) in **Table 3.**

**Table 3:** Highlighted clinically oriented ADE frequencies associated with inhaled corticosteroids.

**ADE Description**	**Budesonide (150–2000 mcg/day)**		**Fluticasone Propionate (200–400 mcg/day**		**Mometasone furoate (200–800 mcg/day)**		**Belcomethasone dipropionate (336mcg/day-1008.3 mcg/day)**		**Ciclesonide (40–160 mcg/day)**		**Inhaled Corticosteroids NOS**	
	% range ADE reported (*n, sample size of study)	Reference Studies	% range ADE reported (n)	Reference Studies	% range ADE reported (n)	Reference Studies	% range ADE reported (n)	Reference Studies	% range ADE reported (n)	Reference Studies	% range ADE reported (n)	Reference Studies
**Gastrointestinal**												
Vomiting	6.06 (198)–15.9(44)	17, 32	3 (102)	34	NR		NR		NR		NR	
**Respiratory**												
Asthma—Death	NR¶		0 (3101)	57	NR		NR		NR		NR	
Asthma—Hospitalization	NR		0.7 (3101)	57	NR		NR		NR		NR	
Asthma—Intubation	NR		0 (3101)	57	NR		NR		NR		NR	
Asthma Exacerbation	2 (198)–9.5 (63)	18, 17, 32, 39	8 (102)–21.8 (257)	21, 38, 34, 33	0 (13)–7.7 (13)	42	19.1 (173)	21	NR		NR	
Bronchitis/Bronchospasm	11.2 (447)–16.3 (1004)	32, 31, 39	2 (257)–14.1 (170)	21, 38, 33	NR		11.6 (173)	21	NR		NR	
Pneumonia	0 (48)–49.6 (119)	18, 17, 23, 39	2.3 (87)–49.1 (114)	21, 23, 38	NR		5.8 (173)	21	NR		NR	
Respiratory Tract Infection	43.8 (1004)–57.9 (447)	17, 32, 31, 39	3.1 (257)	33	NR		10.4 (173)	21	NR		NR	
**Central Nervous System**												
Headache	8.6 (1004)–11.4 (447)	32, 31, 39	20 (1020)	34	0 (13)–40.7 (78)	36, 42	30.9 (81)	36	NR		NR	
**Renal/electrolyte**												
Hypercalcuria	16 (25)	15	NR		NR		NR		NR		43.8 (32)	14
Hypertension	NR		0.4 (257)	33	NR		NR		NR		NR	
**Psychiatry**												
Mood Disorder	NR		3 (102)	34	NR		NR		NR		NR	
Suicidal Behavior	0 (1004)	39	NR		NR		NR		NR		NR	
Unusual Behavior	0 (447)	31	NR		NR		NR		0 (219)–0.5 (221)	41	NR	
**Musculoskeletal/Trauma**												
Accident or Injury	5.6 (198)–16.1 (447)	32, 31, 39	0.4 (257)	33	NR		NR		NR		NR	
Fracture	1 (1004)–2.5 (198)	32, 39	1.2 (87)	38	NR		NR		NR		NR	
**Ear, Nose & Throat**												
Dysphonia	10 (259)	22	NR		NR		11.8 (380)	22	NR		NR	
Hoarseness	11.58 (259)	22	1 (471)–1.2 (257)	19, 33	NR		15.8 (380)	22	0 (219)	41	NR	
Oral Candidasis	0 (63)–10.9 (259)	18, 22, 23, 32, 31	1 (102)–3 (471)	19, 23, 34, 33	4 (74)–4 (78)	36	4 (81)–10.8(380)	22, 36	0 (219)	41	NR	
Tooth Disorder	12.5 (48)–15.9 (33)	17	NR		NR		NR		NR		NR	
**Ophthalmology**												
Cataract	NR		0.2 (432)	19	NR		NR		NR		3.2 (95)	13
**Endocrine**							NR		NR			
Adrenal Suppression—Decreased serum cosyntropin response from normal to subnormal	6.3 (16)–14.3 (28)	17, 50	4.4 (68)	50	NR		NR		42.9 (7)	50	NR	
Adrenal Suppression - Decreased AM Cortisol Level (after 52 weeks)	NR		0.01 (471)	19	NR		NR		NR			
Adrenal Suppression—Urine Free Cortisol Decrease	1.6 (63)	18	13–27 (471)	19	NR		NR		NR		NR	
Diabetes/Elevated Glucose	0.5 (198)	32	<1% (102)	34	NR		NR		NR		NR	
Growth Suppression (<20 mm/year)	NR		1.7 (471)	19	NR		NR		NR		NR	
Growth Velocity (<3%tile)	NR		28.5 (137)	21	NR		54.3 (140)	21	NR		NR	
Growth Velocity (<10%tile)	NR		38 (137)	21	NR		72.9 (140)	21	NR		NR	
Growth Velocity (<25%tile)	NR		55.5 (137)	21	NR		85.7 (140)	21	NR		NR	
Growth Velocity (<50%tile)	NR		74.5 (137)	21	NR		93.6 (140)	21	NR		NR	
Osteopenia	NR		NR		NR		NR		NR		10.8 (210)–23.8 (307)	27
Osteoporosis	0 (1004)	39	NR		NR		NR		NR		NR	
**Summary**												
Any ADE NOS	83.8 (198)–98 (44)	17, 32	15.8 (114)–57 (102)	23, 34	NR		NR		90 (219)–94.6 (221)	41	NR	
Any Drug-related ADE NOS	NR		4 (471)–23 (87)	19, 38	NR		NR		NR		NR	
Serious ADE NOS	8.3 (447)–13.1 (198)	32, 31, 39	0.7 (3101)–5 (471)	19, 38, 57	1.35 (74)–3.85 (78)	36	1.2 (81)	36	NR		NR	

* n = sample size (i.e. denominator), rather than number of cases reported (i.e. numerator) to indicate study power.

**¶** Not reported (NR) indicates that an ADE not monitored, and should be distinguished from a 0% frequency which indicates that an ADE was monitored for but not found

Growth suppression was reported with fluticasone propionate and beclomethasone propionate.[19, 21] In one study, 2% of participants experienced low growth velocities of <20mm/year when exposed to 100mcg BID inhaled fluticasone propionate over one year.[19] In a second study, growth suppression was described with growth velocity percentiles <50% in 75% of participants exposed to 400 mcg/day inhaled fluticasone propionate over one year, and 94% of participants exposed to 400 mcg/day inhaled belcomethasone propionate over one year.[21]

Adrenal supression was reported with budesonide, fluticasone propionate, ciclesonide.[17–19, 50], as assessed with ACTH simulation tests, AM cortisol levels or urine cortisol excretion. In one study, adrenal function was assessed by comparing serum cosyntropin stimulation (ACTH) levels before and after 12 weeks of exposure to placebo and budesonide at both 0.5mg and 1mg.[17] A range of 12% -14% patients experienced a decreased serum cosyntropin (ACTH) stimulation response level from normal baseline to subnormal after 12 weeks. A range of 7%–18% had an increased serum cosyntropin response from subnormal to normal levels. In the second study, decreased cosyntropin response was detected in 6%, 4.% and 43% of patients exposed to budesonide, fluticasone propionate and ciclesonide, respectively. In a third study, 24-hour urine free cortisol was measured in patients exposed to budesonide at three time points: baseline, 12 and 26 weeks after exposure.[18] 2% of patients exposed to budesonide had an abnormally low urinary cortisol. Of note, 8% patients experienced shifts from normal to low urine free a cortisol, although these were not considered adverse events. The final study also examined urine free cortisol, measured from 12-hour overnight samples at weeks 0, 28 and 52 in patients exposed to fluticasone propionate.[19] Urine free cortisol decreased by ≥ 30% in 27% patients and ≥ 50% in 13% patients by 52 weeks of exposure.

### Secondary outcomes

We report severe ADE case descriptions and their frequencies in **Table 4.** Only ADE labelled by authors as serious or severe ADE are included in this table. Some described ADE such as asthma related intubation and ICU admission are inherently serious [57], but were not reported as such by the authors and therefore not included in Table 4.

**Table 4:** Severe ADE descriptions and frequency.

Serious ADE Description	Reference Studies	Total number of cases reported in all included studies (n, total patients exposed to medication in included studies where serious ADE is reported)														
		ICS				ICS + LABA		LABA	SABA			Oral beta-agonist		Cromoglycate		Anti-IgE
		Budes	FP	MF	BDP	Budes + Form	FP + Salmet	Form	Salbut	Leva	RAlb	Bamb	Terb	NaCromo	Nedo	Omal
Appendicitis	33, 16, 35, 39	7 (1004)	1 (257)													5 (849)
Cholelithasis	32	1 (198)														
Ulcerative gastritis and cholecystitis	16															1 (225)
Asthma Exacerbation	32,17, 33, 18, 28, 40, 25, 39	59 (1484)	9 (257)			1 (123)			1 (26)		1 (52)					
Bronchospasm	32, 17	6 (198)							1 (44)							2 (624)
Pneumonia	23, 32, 17, 18, 35, 39	14 (1576)				1 (123)										3 (624)
Upper respiratory tract infection	33		1 (257)						1 (44)							
Respiratory Tract Infection	17, 25 33	2 (141)	2 (257)													
Urology complaints NOS	23	2 (233)														
Dehydration	32	2 (198)														
Drug interaction, overdose or trauma	23	1 (233)														
Cervical Whiplash	16															1 (225)
Fracture	38, 39	10 (1004)	1 (87)													
Injury	39	11														
Chronic otitis media	16															1 (225)
Pharyngitis	39	9 (1004)														
Sickle cell crisis	18	1 (187)														
Urticaria	16															1 (225)
Skin disorder NOS	23		1 (114)													
Anxiety/Nervousness	40									1 (58)						
Severe Restlessness												17 (104)	9 (51)			
Seizure	33		1 (257)													
Nephrotic syndrome	33		1 (257)													
Pharyngitis	33		1 (257)													
Serious ADE NOS	16, 19, 37, 32, 31, 39, 35, 36, 38, 57	161 (1649)	48(3659)	4 (152)	1 (81)		27 (3107)	35 (1637)	30 (1653)				5 (88)	9 (154)		55 (849)

Bud, Budesonide; FP, Fluticasone propionate; Mometasone furoate, MF; BDP, Beclomethasone dipropionate; Form, Formeterol; Salmet, Salmeterol; Salbut, Salbutamol; Levalb, Levalbuterol; RAlb, Racemic Albuterol; Bamb, Bambuterol; Terb, Terbutaline; NaCromo, Sodium cromoglycate; Nedo, Nedocromil sodium; Omal, Omalizumab

Authors reported severe ADE were in seven medication classes: ICS, ICS_ LABA, LABA, SABA, oral beta-agonists, cromoglycates and anti-IgE (Table 4). For all medication classes serious ADE frequency ranged from 1% (fluticasone+salmeterol) to 7% (omalizumab). Definitions of severe or serious ADE varied with study. In one budesonide study, severe ADE were defined as: “death, permanent or severe disability, events requiring hospitalization, life-threatening events, any congenital abnormality or cancer”.(31) In the other budesonide studies (32, 39), ADE were labeled as severe by the authors without a described definition or method for determining severity. In both fluticasone propionate studies, severe ADE were labeled by authors without a description of the method used to determine severity. (19, 31) With the one SABA/LABA study commenting on serious ADE, the study defined severe ADE as asthma exacerbations requiring a course of oral corticosteroids lasting ≥ 5 days, emergency treatment with nebulized epinephrine, injected corticosteroid, or hospitalization (37).

There were no confirmed pediatric deaths observed in any study. Deaths were encountered in one study on salbutamol and formeterol that combined adult and pediatric data with a separate pediatric subgroup analysis but did not report further details on pediatric severe SABA or LABA ADE. <1% (11/8938) of patients exposed to salbutamol died in this study, but it is unclear how many, if any, of these deaths occurred in children. The same study also reported proportions of severe LABA ADE, with a prevalence of 2% (n = 1637) in patients exposed to formeterol.(37) <1% (13/8924) of patients exposed to formeterol died; however it is unclear how many deaths, if any, occurred in children. Causality, as judged by the study’s authors, was unlikely related to medication exposure.

The quality of all included studies was assessed using the Smyth Adapted ADE tool (**S2 Table**). The study design was clearly reported in 39 studies. Sixteen studies described detailed methods to identity ADE, collect data, and provided clear descriptions of the individuals who identified AEs. Three studies clearly described a process to determine ADE causality [25, 31, 39], but no studies utilized a standardized method for assessing causality such as the WHO-UMC [60] or Naranjo [61] scales [62]. Nine studies described a process to determine severity of ADE [23, 30–33, 36, 42], but no studies utilized a standardized method (e.g. NCC MERP for medical errors [63], or U.S. FDA definitions[64]). Finally, no included studies provided data on preventability.

The Cochrane risk of bias assessment was completed on 29 studies (**S2 Table**). Overall risk of bias was low in 11 applicable studies, unclear in 9 and high in 9. The Newcastle-Ottawa Scale was completed on 13 applicable studies, with weaknesses identified in selection and comparability. Cochrane risk of bias and Ottawa Newcastle Scale was not completed in 3 studies due to presentation as an abstract, and in 1 study because of its quasi-experimental design[33].

## Discussion

To our knowledge, our systematic review of literature is unique in its scope of including the full range of asthma medications currently used in children. Our review provides a description of published asthma medication ADE and their frequencies, organized by medication class and organ system. Despite their frequency of use in children, we found a relative paucity of studies examining asthma medication ADE. The majority (60%) of included results focus on ICS, with 94 ADE reported, affecting 13 organ systems including adrenal and growth suppression. The included studies also reported some severe ADE, including 23 deaths. 13 deaths in a LABA study sample both adult and pediatric participants; however due to lack of further reporting we are uncertain if any of these deaths occurred in children.

Prior to our study, the largest review with similar scope was a systematic review of literature examining rates of pediatric asthma medication ADR in clinical trials only. In this study, a similar paucity of data relative to frequency of asthma medication is described, with 12 studies identified in SABA, LABA, ICS and combination medications. In addition, similar to our study, only a few serious ADR are reported, although authors are similarly skeptical of underreporting due to high drop out rates from ADR, heavy pharmacological sponsorship and lack of standard scale for assessing severity. Only one overlapping study [43] was included in our systematic review. This difference in included studies is likely due to a dramatically different search strategy that excluded US trials, as well as inclusion criteria that did not require asthma ADE to be the primary objective. Unlike our study, they found the majority (78%) of studies examined leukotriene receptor antagonists in boys between 6–11 years old, with the most common ADR: asthma exacerbation, respiratory tract infection, cough, fever and headache.

The same authors also published a follow-up retrospective review of pediatric ADR following asthma medication usage reported to a post-market, phamacoviligance network, Vigibase, between 2007 and 2011.[59] Included medication classes were ICS, SABA, LABA, combination ICS+LABA, leukotriene antagonists, and IV SABA. Consistent with our overall findings, ICS generated the greatest number of cases of ADR (46%), although SABA was the drug class associated with the most number of ADR descriptions (21%). In contrast to our overall findings, along with the author’s earlier systematic review[65], this study reported that the majority (85%) of ADR in were classified as “severe”, as per International Centre for Harmonisation of Technical Requirements for Pharmaceuticals for Human Use severity (E2A) criteria, including 6 deaths. Serious ADE included accidental exposure/incorrect dosages, tachycardia, respiratory failure, adrenal insufficiency and aggression. Deaths included disseminated intravascular coagulation (budesonide), severe diarrhea (fluticasone+salmeterol), wheezing exacerbation (fluticasone+salmeterol), drug toxicity (montelukast), premature infant with polyhydramnios (montelukast) and respiratory failure (salbutamol). Although causality and potential for recall bias is high, this contrast in findings likely resulted from the study’s unique methodology as a retrospective database review that captured rare post-market ADR, and eliminated potential publication bias associated with pharmaceautical sponsored studies. Unfortunately, this study design allows for ADE signal generation, but more risk estimation, given an inability to determine patient exposure.

### Inhaled corticosteroids and endocrine/metabolic ADE

While our study is unique as a comprehensive systematic review of pediatric asthma medication ADE frequency, several focused reviews on individual drug classes and ADE have been published. In particular, there are several published reviews on the endocrine and metabolic ADE of ICS: impaired growth and adrenal suppression [66–75] In regards to growth suppression, several reviews consistently describe small dose-dependent impairments to childhood growth velocity, as well as a controversy on the effects of this decreased growth velocity on final adult height, with some ICS associated with permanent decreased adult height, but others no change. [68–71, 74–76] Most notably, a recent Cochrane meta analysis of 25 trials involving 8471 children, found a mean decrease of 0.48cm/year in linear growth velocity and 0.61cm decrease from baseline height associated with regular use of all ICS at low and medium doses for one year but was extinguished in subsequent treatment years.[77] Our observation of decreased growth velocity and suppression in children exposed to flucticasone propionate and belcomethasone is in keeping with published literature.

With regards to adrenal function, our study found evidence of adrenal suppression associated with budesonide and fluticasone proprionate use, in the form of decreased urine free cortisol and decreased cosyntropin (ACTH) stimulation test results from normal to subnormal post-exposure. These results are also consistent with prior focused reviews that describe adrenal suppression with ICS, [69, 71, 75], including a recent systematic review of inhaled corticosteroids in both pediatric and adult studies which found dose-response decreased urine free cortisol in patients exposed to beclomethasone (8.4%/100mcg), fluticasone (3.2%/100mcg) and budesonide (3.1%/100mcg), but not ciclesonide.[78] Our findings also support the 2016 position statement from the Canadian Society of Allergy and Clinical Immunology recommending physicians to screen for adrenal suppression in children receiving high dose ICS for more than 6 months, or vulnerable children on medium dose ICS. [79]

### LABA and death

Aside from these reviews on endocrine and metabolic ICS ADE, there have been several reviews on severe ADE associated with LABA. In 2003, SMART (Salmeterol Multicentre Asthma Research Trial), a large randomized placebo-control trial of adults with asthma was prematurely terminated due to concerns with increased mortality from asthma-related events in patients treated with LABA monotherapy, but not combination LABA + inhaled corticosteroid therapy.[80] The results were submitted to the FDA and subsequent studies found similar results, resulting in a public health advisory against LABA monotherapy for both adults and children.[81] A meta-analysis of FDA data in 2008 revealed children 4–11 years old were at highest risk of serious asthma-related events, particularly hospitalization, albeit with no reported deaths.[82] Most recently, a Cochrane review of LABA safety in children[83] did not find a clear association of LABA monotherapy with death, but did find an increased odds ratio of non-fatal severe adverse events in children exposed to formeterol monotherapy, but not salmeterol. We observed asthma exacerbations as the most frequent ADE associated with LABA, although our results do not indicate what proportion required admission. Our observed 2% proportion of severe ADE, with no confirmed deaths, fits with prior findings.

### Leukotriene antagonists and neuropsychiatric ADE

In recent years, there has been an increased focus on neuropsychiatric ADE associated with leukotriene antagonists, owing to a 2008 US FDA alert on possible neuropsychiatric ADE, such as suicide, associated with LTRA. This FDA alert was based on a report generated by the US based MedWatch pharmacoviligence system associating suicidal patients and their medications. Subsequently, an FDA sponsored review was conducted in 2009 of 116 adult and pediatric studies in Merck trials of monteleukast. There were no reports of completed suicide, and rare possible suicidality-related ADE were comparable to controls.[84] Additional reviews of literature demonstrated limited published evidence from well designed studies of neuropsychiatric ADE with LTRA, with medication alerts continuing to be driven by pharmacovigilance case reports.[85, 86] Most recently, a 2016 review of pediatric psychiatric disorders associated with Montelukast using the aforementioned VigiBase demonstrated age-variant neuropsychiatric ADEs, with infants and children developing sleep disturbances and adolescents depression/anxiety and psychotic reactions.[87] Our study results with 36% of ADE affecting the neuropsychiatric system is consistent with these findings. In particular, similar to literature, we found that the 4-11y cohort included in our review, presented with behaviour changes such as sleep disorder, fatigue, hallucination, headache, hyperkinesis or anxiety, but no suicidality.

### Study limitations

As ADE frequency was our primary outcome, we collected only categorical data (i.e. ADE occurrence) and did not collect continuous data such as growth velocity (cm/year). Thus, we excluded 52 potential studies at full text screen due to lack of extractable data as we were unable to comment on the magnitude of ADE, such as degree of growth impairment and adrenal suppression with ICS.

However, the most substantial limitation of this review stems from concerns with methodological quality in the identified studies, both with study design and with identification and reporting ADE. The majority of studies (80%) were RCT, and 64% of these studies had an unclear or high risk of bias. In addition, 80% of the included studies were industry funded, with the potential for conflict of interest with respect to the reporting of ADE. Also, the majority of studies were small (median n = 198), focused on short-term outcomes (median = 6 months) and did not provide ADE data for a placebo group (63%). These deficiencies may have limited our review’s power to detect important rare and long-term outcomes such as death in children exposed to LABA, and suppression of adult height with inhaled corticosteroids. Furthermore, given the predominance of ADE studies focused on ICS (60%) that were based in an outpatient setting (80%), our ability to comment on the frequency of ADE for other drug classes and for asthma medication use in acute care (emergency/inpatient/ intensive care unit) settings is limited.

We also identified concerns with the methodology used to identify and describe ADE. Although there is no universally accepted tool for assessing the quality of ADE reporting, we adapted a previously published tool used in a systematic review on Pediatric ADE.[9] Applying this tool, we found several methodological and reporting issues. Over 80% of included studies lacked a standardized means to detect ADE, a standard definition of “severe” ADE, or causality and preventability assessments. Several ADE were self-reported by participants, were lab abnormalities that may not be clinically important (which is contrary to the definition of ADE), or were labeled as ADE without a clear definition. The determination of “severe” ADE was also not standardized or clearly reported. For example, the ADE labeled by authors as “severe” for ICS included abnormal liver enzymes [3], treatment failures/asthma exacerbations, and remote events unlikely to be related to medication use such as accidents. The lack of standardized causality and preventability assessments, combined with a lack of data on ADE’s in placebo groups, makes it difficult to establish the true frequency of ADE associated with asthma medications.

## Conclusions

Utilizing a rigorous study design, we conducted a broad-based systematic review on ADE associated with asthma medications in children. The results of this review, including a comprehensive summary of ADE frequency, categorized by organ system and drug class, provides a basis for ongoing medication safety monitoring and future prospective studies on asthma medication safety. A key finding from our review was the identification of substantial methodological issues with respect to both study design and the identification and reporting of ADE in the existing literature. These concerns highlight the need for further research on asthma medication ADE in children. We advocate that future studies utilize a standardized methodology to identify ADE, characterize their severity, and assess causality and preventability.

## Supporting information

S1 Protocol Original systematic search protocol. (TIFF)

S2 Protocol Updated systematic search protocol. (TIFF)

S3 Protocol Article screening protocol for inclusion. (TIFF)

S1 Table Index of ADE Descriptions and frequencies associated with common asthma medications. (DOCX)

S2 Table Quality assessments of included studies. (DOCX)

S1 Checklist PRISMA checklist. (DOC)
