# Supplementary figures and images for: A systematic review of adverse drug events associated with administration of common asthma medications in children
Source: PLoS One. 2017 Aug 9;12(8):e0182738. doi: 10.1371/journal.pone.0182738 (PMC5549998; doi:10.1371/journal.pone.0182738)

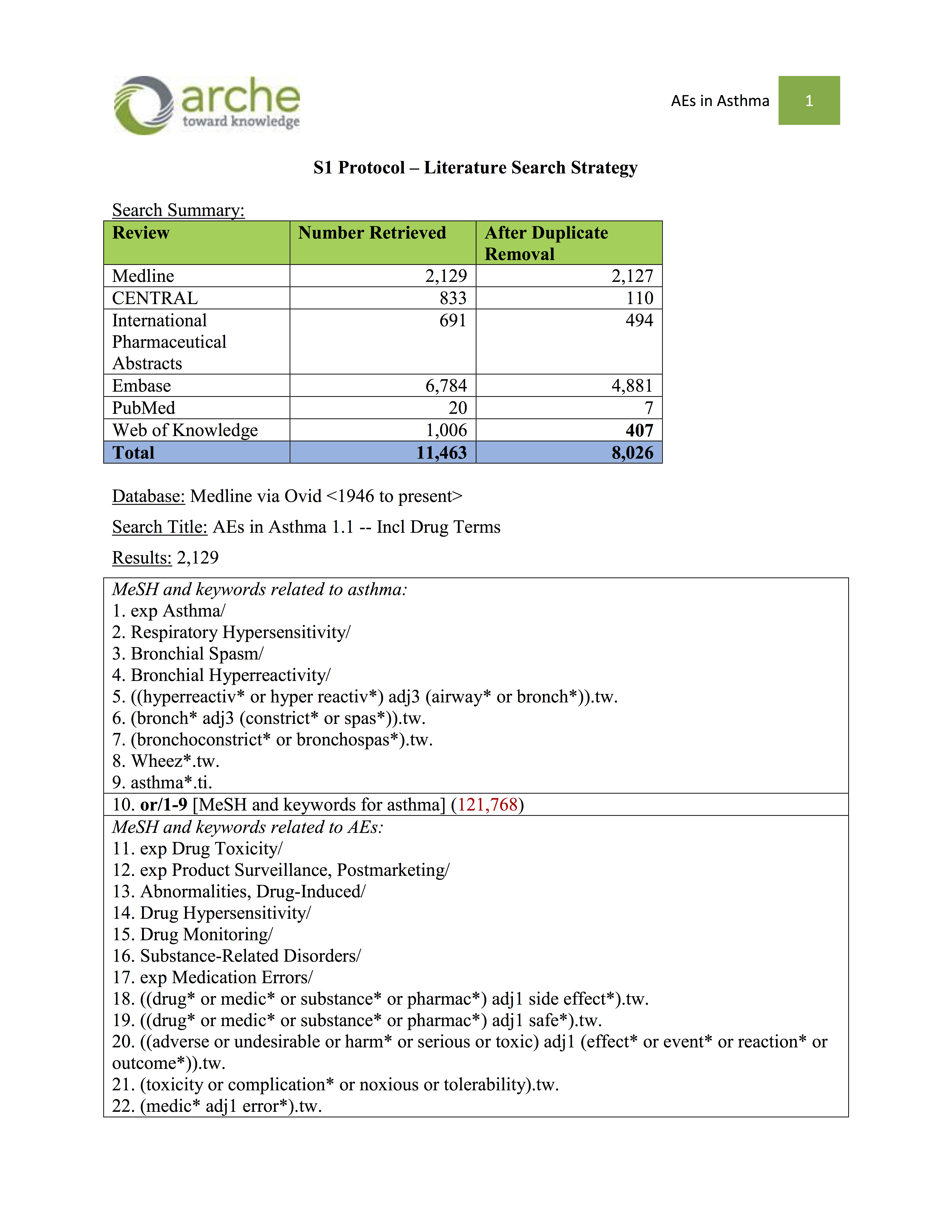

Supplement: S1 Protocol — (TIFF) [file pone.0182738.s001.tiff]

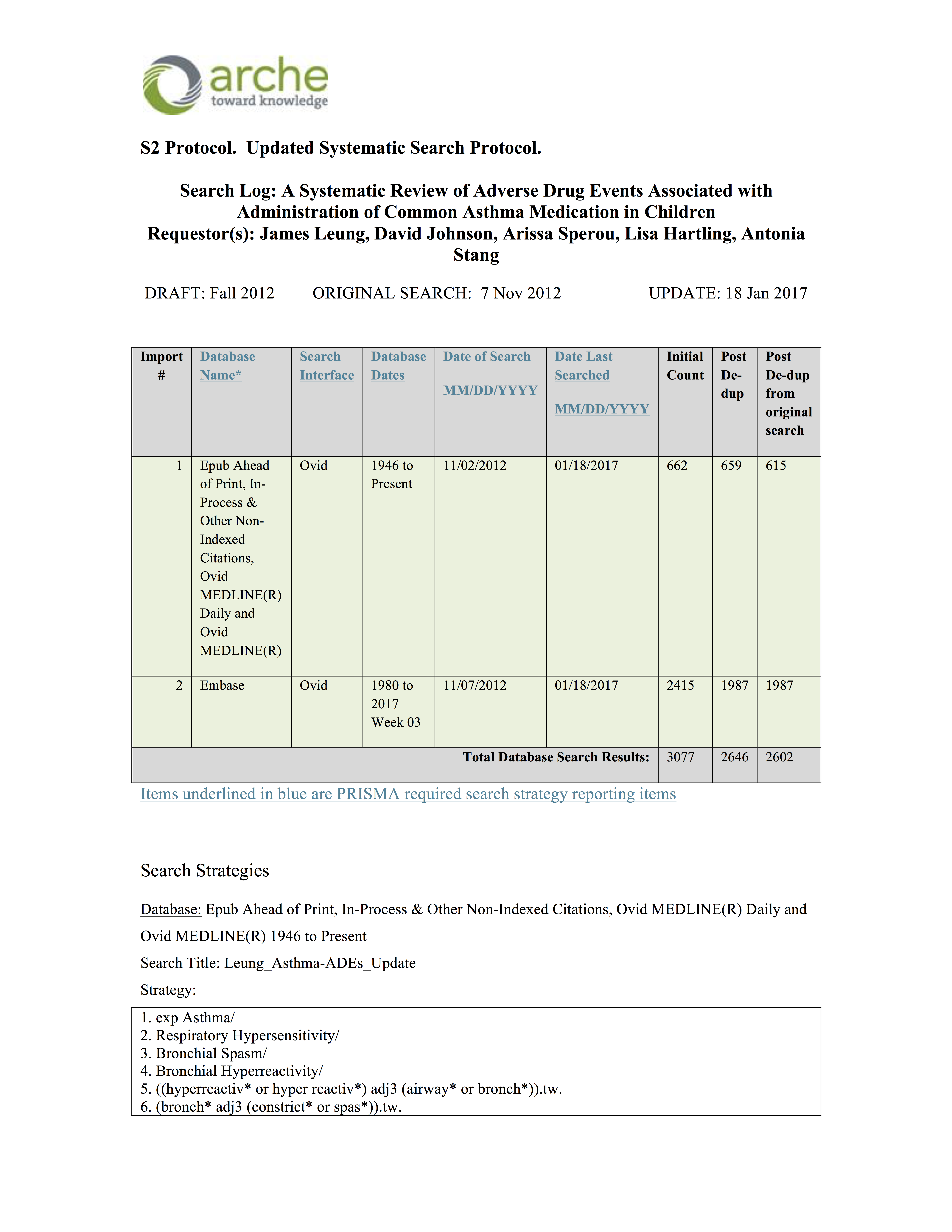

Supplement: S2 Protocol — (TIFF) [file pone.0182738.s002.tiff]

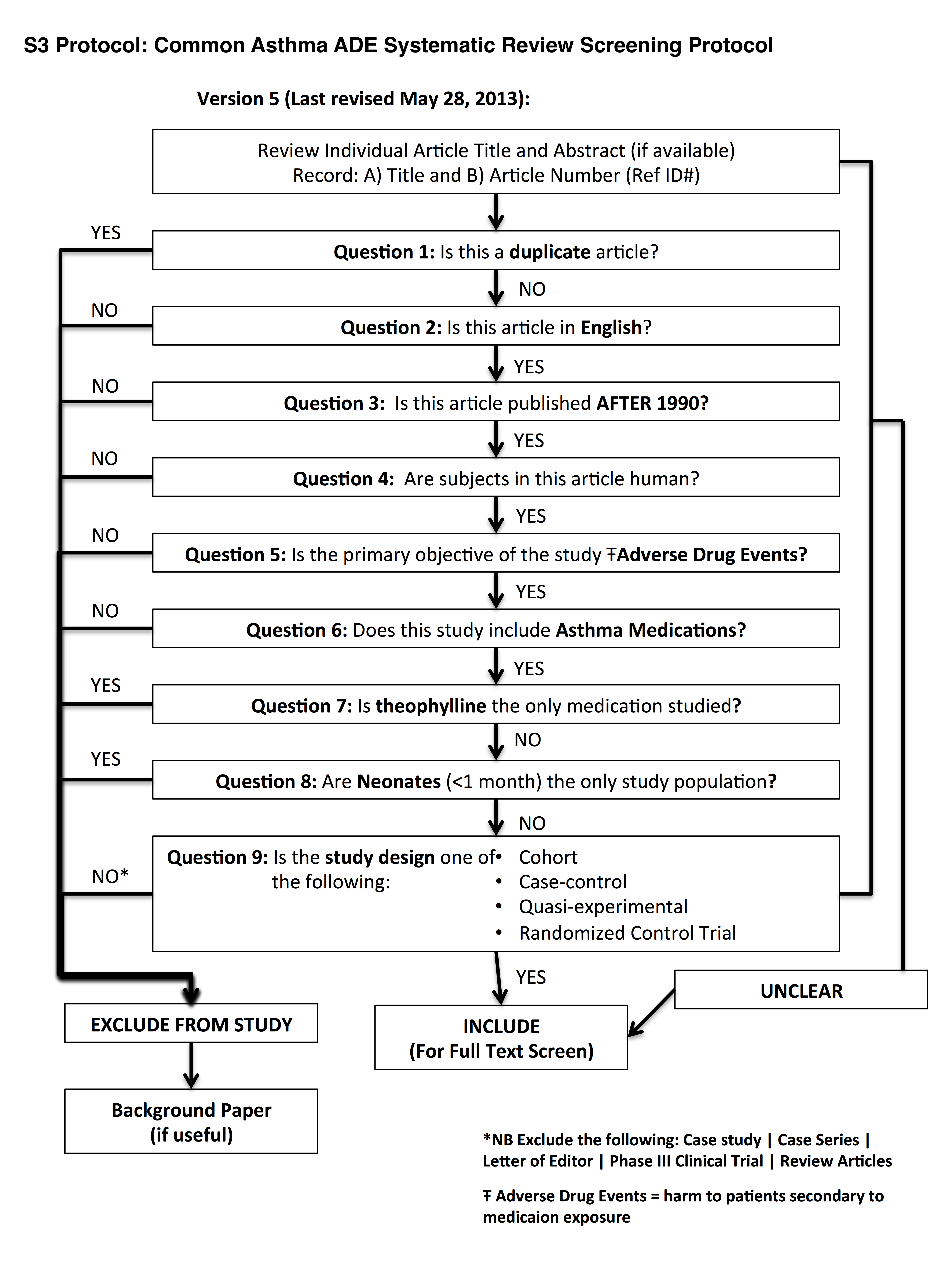

Supplement: S3 Protocol — (TIFF) [file pone.0182738.s003.tiff]
