# Supplementary material for: A systematic review of adverse drug events associated with administration of common asthma medications in children
Source: PLoS One. 2017 Aug 9;12(8):e0182738. doi: 10.1371/journal.pone.0182738 (PMC5549998; doi:10.1371/journal.pone.0182738)
Supplement: S1 Table — (DOCX) [file pone.0182738.s004.docx]

**S1 Table. Index of ADE Descriptions and Frequencies Associated with Common Asthma Medications**

**Inhaled Corticosteroids (ICS)**

| ADE Description | Budesonide (150 – 2000 mcg/day) | | Fluticasone Propionate (200 – 400 mcg/day | | Mometasone furoate (200 – 800 mcg/day) | | Belcomethasone dipropionate (336mcg/day-1008.3 mcg/day) | | Ciclesonide (40 – 160 mcg/day) | | Inhaled Corticosteroids NOS | |
| --- | --- | --- | --- | --- | --- | --- | --- | --- | --- | --- | --- | --- |
|  | % range ADE reported (*n, sample size of study) | Reference Studies | % range ADE reported (n) | Reference Studies | % range ADE reported (n) | Reference Studies | % range ADE reported (n) | Reference Studies | % range ADE reported (n) | Reference Studies | % range ADE reported (n) | Reference Studies |
| Gastrointestinal |  |  |  |  |  |  |  | |  |  |  |  |
| Abnormal Liver Enzymes (AST) | 0.5 (198) | 32 | NR |  | NR |  | NR |  | NR |  | NR |  |
| Appendicitis | 0.7 (1004) | 39 | 0.39 (257) | 33 | NR |  | NR |  | NR |  | NR |  |
| Cholelithiasis | 0.5 (198) | 32 | NR |  | NR |  | NR |  | NR |  | NR |  |
| Diarrhea | 0 (63) – 11.3 (44) | 18, 17 | NR |  | 0 (13) – 8.3 (12) | 42 | NR |  | NR |  | NR |  |
| Gastroenteritis | 8.1 (1004) – 9.8 (447) | 31, 39 | 1.6 (257) – 2.3 (87) | 38, 33 | NR |  | NR |  | NR |  | NR |  |
| GI discomfort | 0 (63)– 7.6 (119) | 18, 23 | 5 (102) | 34 | 0 (13) – 7.7 (13) | 42 | NR |  | NR |  | NR |  |
| Vomiting | 6.06 (198) – 15.9 (44) | 17, 32 | 3 (102) | 34 | NR |  | NR |  | NR |  | NR |  |
| Gastrointestinal Symptoms NOS | NR ¶ |  | 10.53 (114) | 23 | NR |  | NR |  | NR |  | NR |  |
| Respiratory |  |  |  |  |  |  |  | |  |  |  |  |
| Asthma - Death | NR |  | 0 (3101) | 57 | NR |  | NR |  | NR |  | NR |  |
| Asthma Exacerbation | 2 (198) – 9.5 (63) | 18, 17, 32, 39 | 8 (102) – 21.8 (257) | 21, 38, 34, 33 | 0 (13) – 7.7 (13) | 42 | 19.1 (173) | 21 | NR |  | NR |  |
| Asthma - Hospitalization | NR |  | 0.7 (3101) | 57 | NR |  | NR |  | NR |  | NR |  |
| Asthma - Intubation | NR |  | 0 (3101) | 57 | NR |  | NR |  | NR |  | NR |  |
| Bronchitis/bronchospasm | 11.2 (447) – 16.3 (1004) | 32, 31, 39 | 2 (257) – 14.1 (170) | 21, 38, 33 | NR |  | 11.6 (173) | 21 | NR |  | NR |  |
| Cough | 1.6 (63) – 24.3 (259) | 18, 17, 22, 39 | 1.6 (257) – 5.3 (170) | 19, 21, 34, 33 | 7.7 (13) – 8.3 (12) | 42 | 8.1 (173) – 50.3 (380) | 21, 22 | NR |  | NR |  |
| Pneumonia | 0 (48) – 49.6 (119) | 18, 17, 23, 39 | 2.3 (87) – 49.1 (114) | 21, 23, 38 | NR |  | 5.8 (173) | 21 | NR |  | NR |  |
| Respiratory Tract Infection | 43.8 (1004) – 57.9 (447) | 17, 32, 31, 39 | 3.1 (257) | 33 | NR |  | 10.4 (173) | 21 | NR |  | NR |  |
| Rhonchi | 2.1 (48) – 2.3 (44) | 17 | NR |  | NR |  | NR |  | NR |  | NR |  |
| Serious Respiratory System Disorder | 7.8 (1004) | 39 | NR |  | NR |  | NR |  | NR |  | NR |  |
| Upper Respiratory Tract Infection | NR |  | 3 (102) – 17 (102) | 21, 34, 33 | 0 (13) – 48.7 (78) | 36, 42 | 14.5 (173) – 50.6 (81) | 21, 36 | NR |  | NR |  |
| Cardiology |  |  |  |  |  |  |  | |  |  |  |  |
| Chest Pain | 0 (63) | 18 | NR |  | 0 (12) – 15.4 (13) | 42 | NR |  | NR |  | NR |  |
| Increased Heart Rate | 0 (63) | 18 | NR |  | NR |  | NR |  | NR |  | NR |  |
| Ventricular Extrasystoles | 0 (63) | 18 | NR |  | NR |  | NR |  | NR |  | NR |  |
| Chest Symptoms NOS | NR |  | 1 (102) | 34 | NR |  | NR |  | NR |  | NR |  |
| Dermatology |  |  |  |  |  |  |  | |  |  |  |  |
| Bruising | NR |  | 0.4 (257) | 33 | NR |  | NR |  | NR |  | NR |  |
| Dermatitis | NR |  | 2.7 (257) | 33 | 0 (13) – 8.3 (12) | 42 | NR |  | NR |  | NR |  |
| Ezcema | NR |  | 0.4 (257) – 2.3 (87) | 38, 33 | NR |  | NR |  | NR |  | NR |  |
| Fungal Skin Infection | 0.4 (1004) | 39 | NR |  | NR |  | NR |  | NR |  | NR |  |
| Rash | 6.1 (198) – 10.9 (119) | 17, 23, 32 | NR |  | 0 (13) – 7.7 (13) | 42 | NR |  | NR |  | NR |  |
| Skin Symptoms NOS | NR |  | 9.7 (114) | 23 | NR |  | NR |  | NR |  | NR |  |
| Central Nervous System |  |  |  |  |  |  |  | |  |  |  |  |
| Dizziness | NR |  | 0 (102) | 34 | 0 (13) – 8.3 (12) | 42 | NR |  | NR |  | NR |  |
| Headache | 8.6 (1004) – 11.4 (447) | 32, 31, 39 | 20 (1020) | 34 | 0 (13) – 40.7 (78) | 36, 42 | 30.9 (81) | 36 | NR |  | NR |  |
| Hyperkinesis | 0.2 (0.22) | 31 | NR |  | NR |  | NR |  | NR |  | NR |  |
| Seizure | NR |  | 0.4 (257) | 33 | NR |  | NR |  | NR |  | NR |  |
| Neurology Symptoms NOS | NR |  | 0.9 (114) | 23 | NR |  | NR |  | NR |  | NR |  |
| Renal/electrolyte |  |  |  |  |  |  |  | |  |  |  |  |
| Creatinine/BUN Abnormality | 0 (198)– 3.5 (198) | 32 | NR |  | NR |  | NR |  | NR |  | NR |  |
| Dehydration | 1.01 (198) | 32 | NR |  | NR |  | NR |  | NR |  | NR |  |
| Edema | NR |  | NR |  | 0 (13) – 7.7 (13) | 42 | NR |  | NR |  | NR |  |
| Hypercalcuria | 16 (25) | 15 | NR |  | NR |  | NR |  | NR |  | 43.8 (32) | 14 |
| Hypertension | NR |  | 0.4 (257) | 33 | NR |  | NR |  | NR |  | NR |  |
| Potassium Disorder | 0.5 (198) – 1.5 (198) | 32 | 0 (102) | 34 | NR |  | NR |  | NR |  | NR |  |
| Urine Creatinine Decrease | 1.5 (63) | 18 | NR |  | NR |  | NR |  | NR |  | NR |  |
| Urology complication | 0.8 (119) | 23 | 0 (114) | 23 | NR |  | NR |  | NR |  | NR |  |
| Psychiatry |  |  |  |  |  |  |  | |  |  |  |  |
| Fatigue | 0 (63) | 18 | NR |  | NR |  | NR |  | NR |  | NR |  |
| Insomnia/Sleep Disorder | 0 (1004) – 1.59 (63) | 18, 39 | 0 (102) | 34 | NR |  | NR |  | NR |  | NR |  |
| Mood Disorder | NR |  | 3 (102) | 34 | NR |  | NR |  | NR |  | NR |  |
| Suicidal Behavior | 0 (1004) | 39 | NR |  | NR |  | NR |  | NR |  | NR |  |
| Unusual Behavior | 0 (447) | 31 | NR |  | NR |  | NR |  | 0 (219) – 0.5 (221) | 41 | NR |  |
| Musculoskeletal/Trauma |  |  |  |  |  |  |  | |  |  |  |  |
| Accident or Injury | 5.6 (198) – 16.1 (447) | 32, 31, 39 | 0.4 (257) | 33 | NR |  | NR |  | NR |  | NR |  |
| Contusion and Hematoma | NR |  | 3 (102) | 34 | NR |  | NR |  | NR |  | NR |  |
| Fracture | 1 (1004) - 2.5 (198) | 32, 39 | 1.2 (87) | 38 | NR |  | NR |  | NR |  | NR |  |
| MSK Pain | NR |  | 3 (102) | 34 | 0 (13) – 7.7 (13) | 42 | NR |  | NR |  | NR |  |
| Ear, Nose & Throat |  |  |  |  |  |  |  | |  |  |  |  |
| Dysphonia | 10 (259) | 22 | NR |  | NR |  | 11.8 (380) | 22 | NR |  | NR |  |
| ENT Infection | NR |  | 1 (102) – 14.2 (170) | 21, 38, 34 | NR |  | 9.25 (173) | 21 | NR |  | NR |  |
| ENT Pain/Irritation | NR |  | 4.6 (87) – 7 (102) | 34, 38 | 0 (13) – 8.3 (12) | 42 | NR |  | NR |  | NR |  |
| Epistaxis | NR |  | 1 (102) | 34 | 0 (13) – 8.3 (12) | 42 | NR |  | NR |  | NR |  |
| Hearing Impairment | NR |  | NR |  | 0 (13) – 7.7 (13) | 42 | NR |  | NR |  | NR |  |
| Hoarseness | 11.58 (259) | 22 | 1 (471) – 1.2 (257) | 19, 33 | NR |  | 15.8 (380) | 22 | 0 (219) | 41 | NR |  |
| Laryngitis | NR |  | 2.3 (87) | 38 | NR |  | NR |  | NR |  | NR |  |
| Nasal Congestion | NR |  | NR |  | 9 (78) – 23 (74) | 36 | 13.6 (81) | 36 | NR |  | NR |  |
| Oral Candidasis | 0 (63) – 10.9 (259) | 18, 22, 23, 32, 31 | 1 (102) – 3 (471) | 19, 23, 34, 33 | 4 (74) – 4 (78) | 36 | 4 (81) – 10.8 (380) | 22, 36 | 0 (219) | 41 | NR |  |
| Otitis Media | 11.2 (1004) – 47.9 (48) | 17, 32, 31, 39 | NR |  | NR |  | NR |  | NR |  | NR |  |
| Pharyngitis/Tonsillitis | 0 (44) – 28.9 (1004) | 17, 32, 31, 39 | 1.5 (87) – 12.4 (170) | 21, 34, 38, 33 | 0 (13) – 33.8 (74) | 42, 36 | 9.83 (173) – 30.9 (81) | 21, 36 | 17 (219) – 20 (221) | 41 | NR |  |
| Rhinitis | 9.6 (198) – 28.4 (1004) | 17, 32, 31, 39 | 3.5 (257) – 25.3 (170) | 21, 33 | 27 (74) – 37.2 (78) | 36 | 11.6 (173) – 27.2 (81) | 21, 36 | NR |  | NR |  |
| Rhinorrhea | NR |  | NR |  | 0 (13) – 8.3 (12) | 42 | NR |  | NR |  | NR |  |
| Sinusitis | 8.3 (48) – 32.9 (447) | 17, 32, 31, 39 | 0 (102) – 5.9 (170) | 21, 34 | 19.2 (78) – 20.3 (74) | 36 | 6.4 (173) – 13.6 (81) | 21, 36 | NR |  | NR |  |
| Somatitis | NR |  | 0.4 (257) | 33 | NR |  | NR |  | NR |  | NR |  |
| Thirsty Feeling | 19.3 (259) | 22 | NR |  | NR |  | 24.3 (380) | 22 | NR |  | NR |  |
| Tooth Disorder | 12.5 (48) – 15.9 (33) | 17 | NR |  | NR |  | NR |  | NR |  | NR |  |
| ENT Symptoms NOS | 30.3 (119) | 23 | 1 (102) – 50.9 (114) | 23, 34, 33 | NR |  | NR |  | NR |  | NR |  |
| Hematology |  |  |  |  |  |  |  | |  |  |  |  |
| CBC Changes | 0 – 10.1 (198) | 32 | <1% (102) | 34 | NR |  | NR |  | NR |  | NR |  |
| ITP | 0.5 (198) | 32 | NR |  | NR |  | NR |  | 0 – 0.5 (221) | 41 | NR |  |
| Sickle Cell Crisis | 1.6 (63) | 18 | NR |  | NR |  | NR |  | NR |  | NR |  |
| Ophthalmology |  |  |  |  |  |  |  | |  |  |  |  |
| Cataract | NR |  | 0.2 (432) | 19 | NR |  | NR |  | NR |  | 3.2 (95) | 13 |
| Conjunctvitis | 0 (44) – 14.6 (48) | 17, 32 | NR |  | NR |  | NR |  | NR |  | NR |  |
| Ophthalmologic Disorder NOS | 1.5 (198) | 32 | NR |  | 0 (13) – 7.7 (13) | 42 | NR |  | NR |  | NR |  |
| Endocrine |  |  |  |  |  |  | NR | | NR |  |  |  |
| Adrenal Suppression - Decreased serum cosyntropin response from normal baseline to subnormal | 6.3 (16) – 14.3 (28) | 17, 50 | 4.4 (68) | 50 | NR |  | NR |  | 42.9 (7) | 50 | NR |  |
| Adrenal Suppression - Decreased AM cortisol level (after 52 weeks) | NR |  | 0.01 (471) | 19 | NR |  | NR |  | NR |  |  |  |
| Adrenal Suppression - Urine Free Cortisol Decrease | 1.6 (63) | 18 | 13 – 27 (471) | 19 | NR |  | NR |  | NR |  | NR |  |
| Diabetes/Elevated Glucose | 0.5 (198) | 32 | <1% (102) | 34 | NR |  | NR |  | NR |  | NR |  |
| Growth Enhancement (>140 mm/year) | NR |  | 2.8 (471) | 19 | NR |  | NR |  | NR |  | NR |  |
| Growth Suppression (<20 mm/year) | NR |  | 1.7 (471) | 19 | NR |  | NR |  | NR |  | NR |  |
| Growth Velocity (<3%tile) | NR |  | 28.5 (137) | 21 | NR |  | 54.3 (140) | 21 | NR |  | NR |  |
| Growth Velocity (<10%tile) | NR |  | 38 (137) | 21 | NR |  | 72.9 (140) | 21 | NR |  | NR |  |
| Growth Velocity (<25%tile) | NR |  | 55.5 (137) | 21 | NR |  | 85.7 (140) | 21 | NR |  | NR |  |
| Growth Velocity (<50%tile) | NR |  | 74.5 (137) | 21 | NR |  | 93.6 (140) | 21 | NR |  | NR |  |
| Increased serum cosyntropin level from subnormal baseline to normal (in 12 weeks) | 7.1 (28) – 17.7 (17) | 17 | NR |  | NR |  | NR |  | NR |  | NR |  |
| Osteopenia | NR |  | NR |  | NR |  | NR |  | NR |  | 10.8 (210) – 23.8 (307) | 27 |
| Osteoporosis | 0 (1004) | 39 | NR |  | NR |  | NR |  | NR |  | NR |  |
| Weight–low/malnourish(<-3SD) | NR |  | NR |  | NR |  | NR |  | NR |  | 2 (150) | 49 |
| Weight–low/ less malnourish (<-2SD | NR |  | NR |  | NR |  | NR |  | NR |  | 11.3 (150) | 49 |
| Weight–overweight (>2SD) | NR |  | NR |  | NR |  | NR |  | NR |  | 18.7 (150) | 49 |
| Weight-obese (>3SD) | NR |  | NR |  | NR |  | NR |  | NR |  | 10.7 (150) | 49 |
| Other |  |  |  |  |  |  |  | |  |  |  |  |
| Abnormal Lab Values NOS | 15.9 (447) | 31 | NR |  | NR |  | NR |  | NR |  | NR |  |
| Allergy and Allergic Reaction | NR |  | 3 (102) | 34 | 0 (12) – 28.2 (78) | 36, 42 | 24,7 (81) | 36 | NR |  | NR |  |
| Bacterial Infection | 17.2 (198) | 32 | NR |  | NR |  | NR |  | NR |  | NR |  |
| Drug interaction, Overdose, and Trauma | 0.8 (119) | 23 | 0 (114) | 23 | NR |  | NR |  | NR |  | NR |  |
| Fever | 11.1 (198) – 27.74 (447) | 18, 17, 32, 31, 39 | 4.7 (170) – 13 (102) | 21, 34 | 0 (13) – 27.0 (74) | 36, 42 | 6.36 (173) – 32.1 (81) | 21, 36 | NR |  | NR |  |
| Infect Bite | NR |  | NR |  | 0 (13) – 8.3 (12) | 42 | NR |  | NR |  | NR |  |
| Influenza | NR |  | 3.11 (257) | 33 | NR |  | NR |  | NR |  | NR |  |
| Lupus | 0.5 (198) | 32 | NR |  | NR |  | NR |  | NR |  | NR |  |
| Vaginal Infection | 0 (1004) | 39 | NR |  | NR |  | NR |  | NR |  | NR |  |
| Varicella | 1 (198) | 32 | NR |  | NR |  | NR |  | NR |  | NR |  |
| Viral Infection | 9.2 (198) – 22.73 (447) | 32, 31 | 3.1 (257) – 11.8 (170) | 21, 33 | 0 (12) – 35.1 (74) | 36, 42 | 7.51 (173) – 35.8 (233) | 21, 36 | NR |  | NR |  |
| Any ADE NOS | 83.8 (198) – 98 (44) | 17, 32 | 15.8 (114) - 57 (102) | 23, 34 | NR |  | NR |  | 90 (219) – 94.6 (221) | 41 | NR |  |
| Any Drug-related ADE NOS | NR |  | 4 (471) – 23 (87) | 19, 38 | NR |  | NR |  | NR |  | NR |  |
| Serious ADE NOS | 8.3 (447) – 13.1 (198) | 32, 31, 39 | 0.7 (3101) – 5 (471) | 19, 38, 57 | 1.35 (74) – 3.85 (78) | 36 | 1.2 (81) | 36 | NR |  | NR |  |

* n = sample size (i.e. denominator), rather than number of cases reported (i.e. numerator) to indicate study power.

**¶** Not reported (NR) indicates that an ADE not monitored, and should be distinguished from a 0% frequency which indicates that an ADE was monitored for but not found.

**Systemic Corticosteroids**

| ADE Description | Prednisolone sodium phosphate (2mg/kg, max 60mg) | | Prednisolone (2mg/kg, max 60mg) | | Oral Corticosteroid NOS (1 to >5 courses) | | |
| --- | --- | --- | --- | --- | --- | --- | --- |
|  | % range ADE reported (*n [exposed to drug in study]) | Reference Studies | % range ADE reported (n) | Reference Studies | % range ADE reported (n) | | Reference Studies |
| Gastrointestinal |  |  |  |  |  |  | |
| Vomiting | 5.4 (92) | 29 | 17.7 (96) | 29 | NR |  | |
| Endocrine |  |  |  |  |  |  | |
| Osteopenia | NR¶ |  | NR |  | 15.5 (15) – 27.3 (436) | | 27 |

* n = sample size (i.e. denominator), rather than number of cases reported (i.e. numerator) to indicate study power.

**¶** Not reported (NR) indicates that an ADE not monitored, and should be distinguished from a 0% frequency, which indicates that an ADE was monitored for but not found.

**Short Acting Beta-Agonists (SABA) – Intermittent Doses**

| ADE Description | Salbutamol (190mcg TID – 360mcg/dose x 6 doses over 3 hours) | | | | Levalbuterol (310mcg TID – 630 mcg/dose x 3 doses over 1 hour) | | Racemic Albuterol 1.25mg (if <33 pounds), 2.5 mg (if >33 pounds) TID | |
| --- | --- | --- | --- | --- | --- | --- | --- | --- |
|  | % range ADE reported (*n [exposed to drug in study]) | | Reference Studies | | % range ADE reported (n) | Reference Studies | % range ADE reported (n) | Reference Studies |
| Gastrointestinal |  |  | | |  |  |  |  |
| Abnormal Liver Enzymes (ALP) | 0 (25) – 3.9 (26) | | 28 | | NR |  | NR |  |
| Diarrhea | 0 (26) – 8 (25) | | 28 | | NR |  | NR |  |
| Metabolism and Nutrition Disorders | ¶NR | |  | | 0 (51) | 24 | NR |  |
| Vomiting | 3.9 (26) – 4 (25) | | 28 | | NR |  | NR |  |
| Gastrointestinal symptoms NOS | NR | |  | | 3.9 (51) | 24 | NR |  |
| Respiratory |  |  | | |  |  |  |  |
| Asthma exacerbation | 0 (25) – 3.85 (26) | | | 28 | 1.7 (58) | 40 | 3.9 (52) | 40 |
| Bronchospasm | 0 (43) – 2.27 (44) | | | 25 | NR |  | NR |  |
| Intubation | 0.8 (1705) | | | 54 | NR |  | NR |  |
| Positive Pressure Ventilation | 0.8 (1705) | | | 54 | NR |  | NR |  |
| Upper Respiratory Tract Infection | 0 (43) – 2.27 (44) | | | 25 | NR |  | NR |  |
| Cardiovascular |  | | |  |  |  |  |  |
| Arrhytmia NOS | 0.8 (1705) | | | 54 | NR |  | NR |  |
| QTc Prolongation | 0 (25) – 12 (25) | | | 25, 28 | NR |  | NR |  |
| Ventricular Ectopy | 2.27 (44) – 6.98 (43) | | | 25 | NR |  | NR |  |
| Premature Ventricular Beats with Unknown Axis | 0 (44) – 2.3 (43) | | | 25 | NR |  | NR |  |
| Supraventricular Ectopy | 0 (44) – 14 (43) | | | 25 | NR |  | NR |  |
| Tachycardia | 13.6 (44) – 14 (43) | | | 25 | NR |  | NR |  |
| Central Nervous System |  | | |  |  |  |  |  |
| Hyperkinesis | 0 (26) – 4 (25) | | | 28 | 0 (58) – 2 (51) | 40 | 1.9 (52) | 40 |
| Tremor | 8 (25) – 11.5 (26) | | | 28 | NR |  | NR |  |
| CNS Symptoms NOS | NR | | |  | 2 (51) | 24 | NR |  |
| Renal/electrolyte |  | | |  |  |  |  |  |
| Hypokalemia | 11 (127) | | | 54 |  |  |  |  |
| Psychiatry |  | | |  |  |  |  |  |
| Anxiety/Nervousness | NR | | |  | 0 (52) – 53.8 (51) | 40 | 0 | 40 |
| Musculoskeletal/Trauma |  | | |  |  |  |  |  |
| MSK symptoms NOS | NR | | |  | 2 (51) | 40 | NR |  |
| Ear, Nose & Throat |  | | |  |  |  |  |  |
| Nasal Congestion | 0 (44) – 3.9 (43) | | | 25 | NR |  | NR |  |
| Rhinorrhea | 0 (44) – 2.3 (43) | | | 25 | NR |  | NR |  |
| Other |  | | |  |  |  |  |  |
| Fever | 2.3 (44) – 7.7 (26) | | | 25, 28 | NR |  | NR |  |
| ICU Admission | 0.6 (1705) | | | 54 |  |  |  |  |
| Infections and Infestations | NR | | |  | 9.8 (51) | 24 | NR |  |
| Any ADE NOS | 34.6 (26) – 52 (25) | | | 28 | 0 (52) – 60.8 (211) | 24, 40 | NR |  |
| Any Drug-related ADE NOS | NR | | |  | 5.88 (51) | 24 | NR |  |
| Serious ADE NOS | 1.8 (1653) | | | 37 | NR |  | NR |  |

* n = sample size (i.e. denominator), rather than number of cases reported (i.e. numerator) to indicate study power.

**¶** Not reported (NR) indicates that an ADE not monitored, and should be distinguished from a 0% frequency which indicates that an ADE was monitored for but not found.

**Short Acting Beta-Agonists (SABA) – Continuous Doses**

| ADE Description | Terbutaline IV (10 mg/kg bolus, followed by 0.4mg/kg/min) | | Salbutamol continuous nebulization (7.5-15mg/hr) | |
| --- | --- | --- | --- | --- |
|  | % range ADE reported (*n [exposed to drug in study]) | Reference Studies | % range ADE reported (n) | Reference Studies |
| Respiratory |  |  |  |  |
| Asthma | ¶NR |  | NR |  |
| Cough | NR |  | NR |  |
| Intubation | NR |  | 0 (1298) | 54 |
| Positive Pressure Ventilation |  |  | 6 (1298) | 54 |
| Cardiovascular |  |  |  |  |
| Arrhythmia NOS | NR |  | 0.5 (1298) | 54 |
| Elevated CK (>150 IU/L) | 58.6 (29) | 20 |  |  |
| Elevated CK-MB | 10.3 (29) | 20 |  |  |
| Elevated Troponin T | 10.3 (29) | 20 | 24 (50) – 36 (64) | 53, 56 |
| Ischaemic Findings on ECG | 69 (29) | 20 | 7.9 (38) – 30 (50) | 53, 56 |
| Elevated Troponin and Ischemic ECG changes | NR |  | 8 (50) | 53 |
| Hypotension - diastolic | NR |  | 66 (50) – 98 (64) | 53, 56, 58 |
| Hypotension - systolic | NR |  | 11 (90) – 16 (64) | 56 |
| QT Prolongation/ Borderline | NR |  | 26 (38) | 56 |
| Tachycardia | NR |  | 94 (90) – 95 (64) | 56 |
| Dermatology |  |  |  |  |
| Herpes Zoster | NR |  |  |  |
| Central Nervous System |  |  |  |  |
| Headache | NR |  |  |  |
| Psychomotor Hyperactivity | NR |  |  |  |
| Renal/electrolytes |  |  |  |  |
| Hypokalemia | NR |  | 12.2 (238) | 54 |
| Lactic acidosis | NR |  | 80.6 (36) | 48 |
| Ear, Nose & Throat |  |  |  |  |
| Allergic Rhinitis | NR |  |  |  |
| Pharyngitis | NR |  |  |  |
| Pharyngolaryngeal Pain | NR |  |  |  |
| Ophthalmology |  |  |  |  |
| Allergic Conjunctivitis | NR |  |  |  |
| Periorbital Edema | NR |  |  |  |
| Other |  |  |  |  |
| Fever | NR |  |  |  |
| ICU Admission | NR |  | 4.1 (1298) | 54 |
| Influenzae | NR |  |  |  |
| Total ADE | NR |  |  |  |

*n = sample size (i.e. denominator), rather than number of cases reported (i.e. numerator) to indicate study power.

**¶** Not reported (NR) indicates that an ADE not monitored, and should be distinguished from a 0% frequency which indicates that an ADE was monitored for but not found.

**Oral Beta Agonist**

| ADE Description | Bambuterol (10 – 20 mg/day) | | Terbutaline sulphate (0.225 mg/kg/day to 7.5 mg /day maximum) | |
| --- | --- | --- | --- | --- |
|  | % range ADE reported (*n [exposed to drug in study]) | Reference Studies | % range ADE reported (n) | Reference Studies |
| Gastrointestinal |  |  |  |  |
| Abnormal liver enzymes (AST) | 80.8 (104) | 30 | 84.3 (51) | 30 |
| Respiratory |  |  |  |  |
| Bronchitis/bronchospasm | 24 (100) | 47 | ¶0 (50) | 47 |
| Respiratory Tract Infection | 30 (100) | 47 | 28 (50) | 47 |
| Central Nervous System |  |  |  |  |
| Headache | 6 (100) | 47 | 9 (50) | 47 |
| Tremor | 2 (100) | 47 | 0 (50) | 47 |
| Renal/Electrolyte |  |  |  |  |
| Potassium Disorder | 0 (104) | 30 | 80.4 (51) | 30 |
| Psychiatry |  |  |  |  |
| Restlessness | 87.50 (104) | 30 | 86.3 (51) | 30 |
| Ear, Nose & Throat |  |  |  |  |
| Otitis Media | 32 (100) | 47 | 42 (50) | 47 |
| Ophthalmology |  |  |  |  |
| Conjunctivitis | 0 (100) | 47 | 19 (50) | 47 |
| Endocrine |  |  |  |  |
| Elevated Glucose | 78.9 (104) | 30 | 0 (50) | 47 |
| Other |  |  |  |  |
| Any ADE NOS | 90 (100) | 47 | 88 (100) | 47 |

*n = sample size (i.e. denominator), rather than number of cases reported (i.e. numerator) to indicate study power.

**¶** Not reported (NR) indicates that an ADE not monitored, and should be distinguished from a 0% frequency which indicates that an ADE was monitored for but not found.

**Long Acting Beta-Agonists (LABA)**

| ADE Description | Arformeterol (7.5 – 15 mcg/dose x 3 doses) | | Formeterol (4.5 mcg/dose PRN) | | Salmeterol xinafolate (42 – 84 mcg/day) | |
| --- | --- | --- | --- | --- | --- | --- |
|  | % range ADE reported (*n [exposed to drug in study]) | Reference Studies | % range ADE reported (n) | Reference Studies | % range ADE reported (n) | Reference Studies |
| Gastrointestinal |  |  |  |  |  |  |
| Metabolism and Nutrition disorders | 0 (52) – 2.5 (40) | 24 | NR |  | NR |  |
| Gastrointestinal Symptoms NOS | 1.9 (52) – 5 (40) | 24 | NR |  | NR |  |
| Respiratory |  |  |  |  |  |  |
| Asthma Exacerbation | NR¶ |  | NR |  | 7.3 (82) – 8.9 (79) | 45 |
| Respiratory Symptoms NOS | 2.5 (40) – 3.9 (52) | 24 | NR |  | NR |  |
| Cardiovascular |  |  |  |  |  |  |
| Cardiovascular-related ADE NOS | NR |  | 1.3 (8924) | 37 | NR |  |
| Central Nervous System |  |  |  |  |  |  |
| Headache | NR |  | NR |  | 3.7 (82) – 3.8 (79) | 45 |
| CNS symptoms NOS | 0 (52) – 2.5 (40) | 24 | NR |  | NR |  |
| Musculoskeletal/Trauma |  |  |  |  |  |  |
| MSK symptoms NOS | 0 (52) – 2.5 (40) | 24 | NR |  | NR |  |
| Ear, Nose & Throat |  |  |  |  |  |  |
| Administration Site Concerns | 0 (40) – 1.3 (52) | 24 | NR |  | NR |  |
| Other |  |  |  |  |  |  |
| Infections and Infestations | 2 (52) – 7.5 (40) | 24 | NR |  | NR |  |
| Lab Abnormalities NOS | NR |  | NR |  | 8.9 (79) – 9.8 (82) | 45 |
| Any ADE NOS | 13.5 (52) - 20 (40) | 24 | NR |  | NR |  |
| Any Drug-related ADE NOS | 3.9 (52) – 5 (40) | 24 | NR |  | NR |  |
| Severe ADE NOS | NR |  | 2.1 (1637) | 37 | 0 (79) – 0 (82) | 45 |

n = sample size (i.e. denominator), rather than number of cases reported (i.e. numerator) to indicate study power.

**¶** Not reported (NR) indicates that an ADE not monitored, and should be distinguished from a 0% frequency which indicates that an ADE was monitored for but not found.

**Combined ICS and LABA**

| ADE Description | Budesonide (320 mcg/day) + Formeterol (9 mcg/day) | | Fluticasone propionate (100-200 mcg/day) + Salmeterol (50-100 mcg/day) | | Mometasone-formoterol (200-800 mcg/day) | |
| --- | --- | --- | --- | --- | --- | --- |
|  | % range ADE reported (*n [exposed to drug in study]) | Reference Studies | % range ADE reported (n) | Reference Studies | % range ADE reported (n) | Reference Studies |
| Gastrointestinal |  |  |  |  |  |  |
| Diarrhea | 0.8 (123) | 18 | 1 (102) | 34 | NR |  |
| GI Discomfort | 0.8 (123) | 18 | 7 (102) | 34 | NR |  |
| Vomiting | ¶NR |  | 5 (102) | 34 | NR |  |
| Respiratory |  |  |  |  |  |  |
| Asthma-Death | NR |  | 0 (3107) | 57 | NR |  |
| Asthma Exacerbation | 13 (123) | 18 | 3 (102) | 34 | NR |  |
| Asthma-Hospitalization | NR |  | 0.9 (3107) | 57 | NR |  |
| Asthma-Intubation | NR |  | 0 (3107) | 57 | NR |  |
| Cough | 0.8 (123) | 18 | 2 (102) | 34 | NR |  |
| Pneumonia | 0.8 (123) | 18 | NR |  | NR |  |
| Upper Respiratory Tract Infection | NR |  | 1 (102) – 10 (102) | 34 | NR |  |
| Cardiovascular |  |  |  |  |  |  |
| Chest Pain | 0.8 (123) | 18 | NR |  | NR |  |
| Ventricular Extrasystoles | 0.8 (123) | 18 | NR |  | NR |  |
| Increased Heart Rate | 0.8 (123) | 18 | NR |  | NR |  |
| Chest Symptoms NOS | NR |  | 3 (102) | 34 | NR |  |
| Central Nervous System |  |  |  |  |  |  |
| Dizziness | NR |  | 0 (102) | 34 | NR |  |
| Headache | NR |  | 20 (102) | 34 | NR |  |
| Renal/Electrolyte |  |  |  |  |  |  |
| Creatinine/BUN Abnormality | 0 (123) | 18 | NR |  | NR |  |
| Potassium disorder | NR |  | <1% (102) | 34 | NR |  |
| Psychiatry |  |  |  |  |  |  |
| Fatigue | 0.8 (123) | 18 | 0 | 34 | NR |  |
| Insomnia/Sleep Disorder | 0 (123) | 18 | NR |  | NR |  |
| Mood Disorders | NR |  | 1 (102) | 34 | NR |  |
| Musculoskeletal/Trauma |  |  |  |  |  |  |
| Contusion and Hematomas | NR |  | 0 (102) | 34 | NR |  |
| MSK Pain | NR |  | 2 (102) | 34 | NR |  |
| Ear, Nose & Throat |  |  |  |  |  |  |
| ENT Infection | NR |  | 4 (102) | 34 | NR |  |
| ENT Pain/Irritation | NR |  | 8 (102) | 34 | NR |  |
| Epistaxis | NR |  | 4 (102) | 34 | NR |  |
| Oral Candidasis | 0 (123) – 1.6 (123) | 18 | 4 (102) | 34 | NR |  |
| Pharyngitis | NR |  | 3 (102) | 34 | NR |  |
| Sinusitis | NR |  | 3 (102) | 34 | NR |  |
| ENT Symptoms NOS | NR |  | 3 (102) | 34 | NR |  |
| Hematology |  |  |  |  |  |  |
| CBC Changes | NR |  | 0 – <1 (102) | 34 | NR |  |
| Sickle Cell Crisis | 0 (123) | 18 | NR |  | NR |  |
| Endocrine |  |  |  |  |  |  |
| Adrenal Insufficiency | NR |  | NR |  | 14.9 (101) | 55 |
| Diabetes/Elevated Glucose | NR |  | <1 (102) | 34 | NR |  |
| Urine Free Cortisol Decrease | 0 (123) | 18 | NR |  | NR |  |
| Other |  |  |  |  |  |  |
| Allergy and Allergic Reaction Fever | NR |  | 2 (102) | 34 | NR |  |
| Fever | NR |  | 5 (102) | 34 | NR |  |
| Any ADE NOS | NR |  | 59 (102) | 34 | NR |  |
| Severe ADE NOS | NR |  | 0.9 (3107) | 57 | NR |  |

*n = sample size (i.e. denominator), rather than number of cases reported (i.e. numerator) to indicate study power.

**¶** Not reported (NR) indicates that an ADE not monitored, and should be distinguished from a 0% frequency which indicates that an ADE was monitored for but not found.

**Leukotriene Antagonist (LTRA)**

| ADE Description | Montelukast (4 – 8 mg/day) | | LRTA NOS |  |
| --- | --- | --- | --- | --- |
|  | % range ADE reported (*n exposed to drug in study) | Reference Studies | % range ADE reported (n) | Reference Studies |
| Gastrointestinal |  |  |  |  |
| Abdominal Pain | NR |  | 0.6 (1024) | 52 |
| Abnormal Liver Enzymes (AST) | 14.3 (7) – 40 (5) | 26 | NR |  |
| Diarrhea | ¶0 (5) – 20 (5) | 26, 43 | NR |  |
| Vomiting | 0 (5) – 14.29 (7) | 26, 43 | NR |  |
| Respiratory |  |  |  |  |
| Asthma Exacerbation | 1.2 (175) – 18.9 (175) | 43 | NR |  |
| Upper Respiratory Tract Infection | 0 (5) – 54.9 (175) | 26, 43 | NR |  |
| Cardiovascular |  |  |  |  |
| Cyanosis | 0 (7) – 20 (5) | 26 | NR |  |
| Dermatology |  |  |  |  |
| Aphthous ulcers | NR |  | 0.2 (1024) | 52 |
| Rash NOS | NR |  | 0.3 (1024) | 52 |
| CNS |  |  |  |  |
| Headache | NR |  | 0.3 (1024) | 52 |
| Hyperkinesis | NR |  | 0.7 (1024) | 52 |
| Seizure | NR |  | 0.2 (1024) | 52 |
| Psychiatric |  |  |  |  |
| Appetite changes | NR |  | 0.1 (1024) | 52 |
| Anxiety/Nervousness | NR |  | 0.4 (1024) | 52 |
| Fatigue | NR |  | 0.4 (1024) | 52 |
| Hallucination | NR |  | 0.4 (1024) | 52 |
| Insomnia/Sleep Disorder | NR |  | 0.1 (1024) | 52 |
| Nyctophobia | NR |  | 0.4 (1024) | 52 |
| Ear, Nose & Throat |  |  |  |  |
| Nasal Congestion | 0 (5) – 14.3 (7) | 26 | NR |  |
| Ophthalmology |  |  |  |  |
| Conjunctivitis | 0 (7) – 20 (5) | 26 | NR |  |
| Other |  |  |  |  |
| Fever | 0 (5) – 13.1 (175) | 26, 43 | NR |  |
| Fungal Genital Infection | 0 (7) – 20 (5) | 26 | NR |  |
| Lab Abnormalities NOS | 2.3 (175) | 43 | NR |  |
| Any ADE NOS | NR |  | 4 (1024) | 52 |
| Serious ADE NOS | 0 (7) – 0 (5) | 26 | NR |  |

*n = sample size (i.e. denominator), as opposed to than number of cases reported (i.e. numerator) as an indication of study power

**¶** Not reported (NR) indicates that an ADE not monitored, and should be distinguished from a 0% frequency which indicates that an ADE was monitored for but not found.

**Anticholinergics**

| ADE Description | Nebulized ipratropium bromide (250 – 500 mcg/dose) + Saline | | Nebulized ipratropium bromide (250 – 500 mcg/dose) + Nebulized Albuterol (0.1 mg/kg) | |
| --- | --- | --- | --- | --- |
|  | % range ADE reported (*n [exposed to drug in study]) | Reference Studies | % range ADE reported (n) | Reference Studies |
| Cardiovascular |  |  |  |  |
| Palpitations | 10 (20) | 44 | 25 (20) – 40 (20) | 44 |
| Central Nervous System |  |  |  |  |
| Tremor | 10 (20) | 44 | 35 (20) – 40 (20) | 44 |
| Ear, Nose & Throat |  |  |  |  |
| Bad Taste | 15 (20) | 44 | 15 (20) – 30 (20) | 44 |
| Dry Mouth | 30 (20) | 44 | 30 (20) – 35 (20) | 44 |
| Ophthalmology |  |  |  |  |
| Blurry Vision | 5 (20) | 44 | 5 (20) – 20 (20) | 44 |

*n = sample size (i.e. denominator), rather than number of cases reported (i.e. numerator) to indicate study power.

**Cromoglycates**

| ADE Description | Sodium cromoglycates (20 mg/day) | | Nedocromil sodium (8 mg/day) | |
| --- | --- | --- | --- | --- |
|  | % range ADE reported (*n exposed to drug in study) | Reference Studies | % range ADE reported (n exposed to drug in study) | Reference Studies |
| Gastrointestinal |  |  |  |  |
| Gastroenteritis | ¶NR |  | 1.1 (88) | 38 |
| Respiratory |  |  |  |  |
| Asthma Exacerbation | NR |  | 17.1 (88) | 38 |
| Bronchitis/Bronchospasm | NR |  | 1.1 (88) | 38 |
| Cough | 1 (154) | 19 | NR |  |
| Pneumonia | NR |  | 1.1 (88) | 38 |
| Dermatology |  |  |  |  |
| Ezcema | NR |  | 0 (88) | 38 |
| Musculoskeletal/Trauma |  |  |  |  |
| Traumatic Fracture | NR |  | 0 (88) | 38 |
| Ear, Nose & Throat |  |  |  |  |
| ENT Infection | NR |  | 0 (88) | 38 |
| ENT Pain/Irritation | NR |  | 8 (88) | 38 |
| Hoarseness | 0 (154) | 19 | NR |  |
| Laryngitis | NR |  | 1.1 (88) | 38 |
| Oral Candidiasis | 0 (154) | 19 | NR |  |
| Pharyngitis | NR |  | 3.4 (88) | 38 |
| Ophthalmology |  |  |  |  |
| Cataract | 0 (154) | 19 | NR |  |
| Endocrine |  |  |  |  |
| Adrenal Suppression | 4 (154) | 19 | NR |  |
| Decreased AM cortisol level (after 52 weeks) | 0 (154) | 19 |  |  |
| Growth Enhancement (>140 mm/year) | 2.6 (154) | 19 | NR |  |
| Growth Suppression (<20 mm/year) | 2.6 (154) | 19 | NR |  |
| Urine Free Cortisol Decrease | 11 (154) – 18 (154) | 19 |  |  |
| Other |  |  |  |  |
| Any Drug-related ADE NOS | NR |  | 20.5 (88) | 38 |
| Serious ADE NOS | 6 (154) | 19 | 5.7 (88) | 38 |

*n = sample size (i.e. denominator), as opposed to than number of cases reported (i.e. numerator) as an indication of study power

**¶** Not reported (NR) indicates that an ADE not monitored, and should be distinguished from a 0% frequency which indicates that an ADE was monitored for but not found.

**Anti-IgE**

| ADE Description | Omalizumab (75 mg q2weeks – 375mg q2weeks) | |
| --- | --- | --- |
|  | % range ADE reported *(n exposed to drug in study) | Reference Studies |
| Gastrointestinal |  |  |
| Abdominal Pain | 4.2 (624) – 16.9 (225) | 16, 35 |
| Abnormal Liver Enzymes (AST or ALT) | 0.2 (624) – 0.3 (624) | 35 |
| Appendicitis | <1 (225) | 16 |
| Diarrhea | 4 (624) – 5.8 (225) | 16, 35 |
| Gastroenteritis | 3.9 (624) – 6.2 (225) | 16, 35 |
| GI Discomfort | 8.9 (225) | 16 |
| Nausea | 2.7 (624) | 35 |
| Ulcerative Gastritis and Cholecystitis | <1 (225) | 16 |
| Vomiting | 7.7 (624) – 8.9 (225) | 16, 35 |
| Respiratory |  |  |
| Bronchitis/bronchospasm | 6.7 (624) | 35 |
| Cough | 11.4 (624) – 20 (225) | 16, 35 |
| Pneumonia | 1 (624) – 2.7 (225) | 16, 35 |
| Upper Respiratory Tract Infection | 6.7 (624) – 47.1 (225) | 16, 35 |
| Dermatology |  |  |
| Dermatitis | 6.2 (225) | 16 |
| Hypersensitivity Reaction | 6.3 (624) | 35 |
| Rash | 3.4 (624) – 6.7 (624) | 16, 35 |
| Urticaria | 3.5 (624) – 4.9 (225) | 16, 35 |
| Central Nervous System |  |  |
| Headache | 20.7 (624) – 42.7 (225) | 16, 35 |
| Renal/Electrolyte |  |  |
| Creatinine Abnormality | ¶0 (624) | 35 |
| Musculoskeletal/Trauma |  |  |
| Arthralgia | 6.2 (225) | 16 |
| Back Pain | 5.3 (225) | 16 |
| Extremity Pain | 1.9 (624) | 35 |
| Fracture | 5.3 (225) | 16 |
| Injury | 8 (225) | 16 |
| Joint Sprain | 1.8 (624) – 6.7 (225) | 16, 35 |
| Trauma | 8.9 (225) | 16 |
| Ear, Nose & Throat |  |  |
| ENT Pain/Irritation | 10.1 (624) | 35 |
| Epistaxis | 3.4 (624) | 35 |
| Nasal Congestion | 5.1 (624) | 35 |
| Otalagia | 3.2 (624) – 7.1 (225) | 16, 35 |
| Otitis Media | 5 (624) – 10.7 (225) | 16, 35 |
| Pharyngitis | 6.1 (624) – 23.6 (624) | 16, 35 |
| Rhinitis | 4 (624) – 16.4 (225) | 16, 35 |
| Sinusitis | 1.9 (624) – 24 (225) | 16, 35 |
| Tonsillitis | 1.3 (624) | 35 |
| Hematology |  |  |
| Bleeding Disorders | 4.2 (624) | 35 |
| Decreased Hemoglobin or Hematocrit | 0.3 (624) | 35 |
| Decreased Neutrophils | 13.1 (624) | 35 |
| Decreased Platelets | 0.6 (624) | 35 |
| Decreased WBC | 0 (624) | 35 |
| Ophthalmology |  |  |
| Conjunctivitis | 1.6 (624) | 35 |
| Other |  |  |
| Anaphylaxis | 0.2 (624) | 35 |
| Fever | 15.1 (624) – 25.3 (225) | 16, 35 |
| Influenzae | 9.8 (624) | 35 |
| Insect Bite | 3.2 (624) | 35 |
| Serum Sickness | 4 (624) | 35 |
| Viral Infection | 4.3 (624) – 31.6 (225) | 16, 35 |
| Any ADE NOS | 92.9 (225) | 16 |
| Serious ADE NOS | 3.4 (624) | 35 |

*n = sample size (i.e. denominator), as opposed to than number of cases reported (i.e. numerator) as an indication of study power

**¶** Not reported (NR) indicates that an ADE not monitored, and should be distinguished from a 0% frequency which indicates that an ADE was monitored for but not found.

**Other**

| ADE Description | Magnesium sulphate IV infusion (50-70mg/kg bolus) followed by 40mg/kg/h infusion x4h | | Chloroflurocarbon propellant + inhaled beclomethasone dipropionate | | Hydrofluoroalkane-134a propellant + inhaled beclomethasone dipropionate | |
| --- | --- | --- | --- | --- | --- | --- |
|  | % range ADE reported (*n [exposed to drug in study]) | Reference Studies | % range ADE reported (n) | Reference Studies | % range ADE reported (n) | Reference Studies |
| Gastrointestinal |  |  |  |  |  |  |
| Nausea and Vomiting | 5.3 (19) | 51 | NR |  | NR |  |
| Respiratory |  |  |  |  |  |  |
| Asthma | ¶NR |  | 0 (63) | 46 | 1.6 (63) | 46 |
| Cough | NR |  | 1.6 (63) | 46 | 1.6 (63) | 46 |
| Dermatology |  |  |  |  |  |  |
| Flushing | 5.3 (19) | 51 |  |  |  |  |
| Herpes Zoster | NR |  | 1.6 (63) | 46 | 0 (63) | 46 |
| Central Nervous System |  |  |  |  |  |  |
| Headache | NR |  | 1.6 (63) | 46 | 0 (63) | 46 |
| Psychomotor hyperactivity | NR |  | 0 (63) | 46 | 1.6 (63) | 46 |
| Ear, Nose & Throat |  |  |  |  |  |  |
| Allergic Rhinitis | NR |  | 1.6 (63) | 46 | 0 (63) | 46 |
| Pharyngitis | NR |  | 1.6 (63) | 46 | 3.2 (63) | 46 |
| Pharyngolaryngeal Pain | NR |  | 0 (63) | 46 | 1.6 (63) | 46 |
| Ophthalmology |  |  |  |  |  |  |
| Allergic Conjunctivitis | NR |  | 0 (63) | 46 | 1.6 (63) | 46 |
| Periorbital Edema | NR |  | 0 (63) | 46 | 1.6 (63) | 46 |
| Other |  |  |  |  |  |  |
| Fever | NR |  | 1.6 (63) | 46 | 0 (63) | 46 |
| Influenzae | NR |  | 0 (63) | 46 | 1.6 (63) | 46 |
| Pain at IV site | 5.3 (19) | 51 |  |  |  |  |
| Any ADE NOS | NR |  | 9.5 (63) | 46 | 12.7 (63) | 46 |
| Serious ADE NOS | 0 (19) | 51 | NR |  | NR |  |

*n = sample size (i.e. denominator), as opposed to than number of cases reported (i.e. numerator) as an indication of study power

**¶** Not reported (NR) indicates that an ADE not monitored, and should be distinguished from a 0% frequency which indicates that an ADE was monitored for but not found.
