# Supplementary material for: A systematic review of adverse drug events associated with administration of common asthma medications in children
Source: PLoS One. 2017 Aug 9;12(8):e0182738. doi: 10.1371/journal.pone.0182738 (PMC5549998; doi:10.1371/journal.pone.0182738)
Supplement: S2 Table — (DOCX) [file pone.0182738.s005.docx]

**S2 Table. Quality Assessments of Included Studies**

| **Smyth Adapted AE Tool** | | | | | | | |
| --- | --- | --- | --- | --- | --- | --- | --- |
|  | **Behbehani**  **2005 (13)** | **Bentur**  **2000 (14)** | **Bentur**  **2003 (15)** | **Berger**  **2003 (16)** | **Berger**  **2005 (17)** | **Berger**  **2010 (18)** | **Bisgaard**  **2004 (19)** |
| **Study Design** |  |  |  |  |  |  |  |
| Clear study design | Yes | Yes | Unclear | Yes | Yes | Yes | NR |
| **Methods to identify AE** |  |  |  |  |  |  |  |
| Detailed methods to identify AE | Yes | NR | NR | Yes | NR | Yes | NR |
| Detailed data collection methods | Yes | Yes | Unclear | Yes | NR | Yes | NR |
| Clear description of individuals identifying AE | Yes | NR | NR | NR | NR | NR | NR |
| **Methods to identify causality** |  |  |  |  |  |  |  |
| Clear description of process to establish causality | NR | NR | NR | NR | NR | NR | NR |
| Standardized methods used to assess causality | NR | NR | NR | NR | NR | NR | NR |
| **Methods to determine preventability** |  |  |  |  |  |  |  |
| Clear description of process used to establish preventability | NR | NR | NR | NR | NR | NR | NR |
| Standardized methods to assess preventability | NR | NR | NR | NR | NR | NR | NR |
| **Methods to determine severity** |  |  |  |  |  |  |  |
| Clear description of process to establish severity | NR | NR | NR | NR | NR | NR | NR |
| Standardized methods to assess severity | NR | NR | NR | NR | NR | NR | NR |
| **Methods to determine type of AE** |  |  |  |  |  |  |  |
| Clear description of process to establish type of AE | NR | NR | NR | NR | Unclear | Yes | NR |
| Standardized methods to assess type of AE | NR | NR | NR | NR | NR | NR | NR |
| **Cochrane Risk of Bias** | | | | | | | |
| **Selection bias -** random sequence generation |  |  |  | Unclear | Unclear | Low | Low |
| **Selection bias** - allocation concealment |  |  |  | Low | Low | Low | Low |
| **Performance bias -** blinding of participants and researchers |  |  |  | Unclear | Unclear | Unclear | Unclear |
| **Detection bias -** blinding of outcome assessment |  |  |  | Unclear | Unclear | Unclear | Unclear |
| **Attrition bias -** incomplete outcome data |  |  |  | Unclear | Unclear | Unclear | Low |
| **Reporting bias -** selective reporting |  |  |  | Unclear | Unclear | Unclear | Unclear |
| **Other bias** |  |  |  | Unclear | Unclear | Unclear | Unclear |
| **Overall Risk of Bias** |  |  |  | **Unclear risk** | **Unclear risk** | **Unclear risk** | **Unclear risk** |
| **Newcastle-Ottawa Scale (NOS)** | | | | | | | |
| Selection^1^ | 4 | 1 | 1 |  |  |  |  |
| Comparability^2^ | 1 | 1 | 0 |  |  |  |  |
| Outcome^3^ | 2 | 2 | 2 |  |  |  |  |
| **Total (out of maximum 9)** | **7** | **4** | **3** |  |  |  |  |
| ^1^Maximum of 4 stars for: representativeness of the exposed cohort; selection of the non-exposed cohort; ascertainment of exposure; and demonstration that outcome of interest was not present at start of study  ^2^ Maximum of 2 stars for: comparability of cohorts on the basis of the design or analysis  ^3^ Maximum of 3 stars for: assessment of outcome; was follow-up long enough for outcome to occur; and adequacy of follow-up of cohorts | | | | | | | |

**S2 Table. Quality Assessments of Included Studies - continued**

| **Smyth Adapted AE Tool** | | | | | | | |
| --- | --- | --- | --- | --- | --- | --- | --- |
|  | **Chiang**  **2000 (20)** | **deBenedictis 2001 (21)** | **Dubus**  **2001 (22)** | **Ferguson**  **2007 (23)** | **Hinkle**  **2011 (24)** | **Kaashmiri**  **2010 (25)** | **Kearns**  **2008 (26)** |
| **Study Design** |  |  |  |  |  |  |  |
| Clear study design | Yes | Yes | Yes | Yes | Yes | Yes | Yes |
| **Methods to identify AE** |  |  |  |  |  |  |  |
| Detailed methods to identify AE | Yes | Yes | Yes | Yes | Yes | Yes | Yes |
| Detailed data collection methods | Yes | Yes | Yes | Yes | Yes | Yes | Yes |
| Clear description of individuals identifying AE | NR | NR | NR | Yes | Yes | Unclear | NR |
| **Methods to identify causality** |  |  |  |  |  |  |  |
| Clear description of process to establish causality | NR | NR | NR | NR | NR | Yes | NR |
| Standardized methods used to assess causality | NR | NR | NR | Unclear | NR | Unclear | Unclear |
| **Methods to determine preventability** |  |  |  |  |  |  |  |
| Clear description of process used to establish preventability | NR | NR | NR | NR | NR | NR | NR |
| Standardized methods to assess preventability | NR | NR | NR | NR | NR | NR | NR |
| **Methods to determine severity** |  |  |  |  |  |  |  |
| Clear description of process to establish severity | NR | NR | NR | Yes | NR | NR | NR |
| Standardized methods to assess severity | NR | NR | NR | Unclear | NR | NR | Unclear |
| **Methods to determine type of AE** |  |  |  |  |  |  |  |
| Clear description of process to establish type of AE | Yes | NR | Yes | NR | NR | NR | NR |
| Standardized methods to assess type of AE | NR | NR | Unclear | NR | NR | NR | NR |
| **Cochrane Risk of Bias** | | | | | | | |
| **Selection bias -** random sequence generation |  | Low |  | Low | Unclear | Low | Low |
| **Selection bias** - allocation concealment |  | Low |  | Low | Unclear | Low | Unclear |
| **Performance bias -** blinding of participants and researchers |  | Unclear |  | Low | Unclear | Low | Low |
| **Detection bias -** blinding of outcome assessment |  | Unclear |  | Unclear | Low | Unclear | Unclear |
| **Attrition bias -** incomplete outcome data |  | Low |  | Low | Unclear | Low | Low |
| **Reporting bias -** selective reporting |  | Low |  | Low | Unclear | Unclear | Unclear |
| **Other bias** |  | Unclear |  | Low | Unclear | Unclear | Unclear |
| **Overall Risk of Bias** |  | **Low risk** |  | **Low risk** | **Unclear risk** | **Low risk** | **Unclear risk** |
| **Newcastle-Ottawa Scale (NOS)** | | | | | | | |
| Selection^1^ | 2 |  | 2 |  |  |  |  |
| Comparability^2^ | 0 |  | 0 |  |  |  |  |
| Outcome^3^ | 3 |  | 3 |  |  |  |  |
| **Total (out of maximum 9)** | **5** |  | **5** |  |  |  |  |
| ^1^Maximum of 4 stars for: representativeness of the exposed cohort; selection of the non-exposed cohort; ascertainment of exposure; and demonstration that outcome of interest was not present at start of study  ^2^ Maximum of 2 stars for: comparability of cohorts on the basis of the design or analysis  ^3^ Maximum of 3 stars for: assessment of outcome; was follow-up long enough for outcome to occur; and adequacy of follow-up of cohorts | | | | | | | |

**S2 Table. Quality Assessments of Included Studies - continued**

| **Smyth Adapted AE Tool** | | | | | | | |
| --- | --- | --- | --- | --- | --- | --- | --- |
|  | **Kelly**  **2008 (27)** | **Kerwin**  **2006 (28)** | **Kim**  **2006 (29)** | **Kuusela**  **2000 (30)** | **Leflein**  **2001 (31)** | **Leflein**  **2005 (32)** | **MacKenzie**  **1994 (33)** |
| **Study Design** |  |  |  |  |  |  |  |
| Clear study design | Yes | Yes | Yes | Yes | Yes | Yes | Yes |
| **Methods to identify AE** |  |  |  |  |  |  |  |
| Detailed methods to identify AE | NR | Yes | Yes | Yes | Yes | Yes | NR |
| Detailed data collection methods | Yes | Yes | Yes | Yes | Yes | NR | NR |
| Clear description of individuals identifying AE | Yes | Yes | Yes | NR | NR | NR | NR |
| **Methods to identify causality** |  |  |  |  |  |  |  |
| Clear description of process to establish causality | NR | NR | NR | NR | NR | Yes | NR |
| Standardized methods used to assess causality | NR | NR | NR | NR | NR | Unclear | NR |
| **Methods to determine preventability** |  |  |  |  |  |  |  |
| Clear description of process used to establish preventability | NR | NR | NR | NR | NR | NR | NR |
| Standardized methods to assess preventability | NR | NR | NR | NR | NR | NR | NR |
| **Methods to determine severity** |  |  |  |  |  |  |  |
| Clear description of process to establish severity | NR | NR | NR | Yes | Yes | Yes | Yes |
| Standardized methods to assess severity | NR | Unclear | NR | Unclear | Unclear | Unclear | Unclear |
| **Methods to determine type of AE** |  |  |  |  |  |  |  |
| Clear description of process to establish type of AE | NR | NR | Yes | NR | NR | NR | NR |
| Standardized methods to assess type of AE | NR | NR | NR | NR | NR | NR | NR |
|  | | | | | | | |
| **Selection bias -** random sequence generation |  | Unclear | Low | Unclear | Low | High | (Quasi-exp) |
| **Selection bias** - allocation concealment |  | Unclear | Low | Unclear | Unclear | High |  |
| **Performance bias -** blinding of participants and researchers |  | Low | Low | Low | High | High |  |
| **Detection bias -** blinding of outcome assessment |  | Unclear | Unclear | Unclear | High | High |  |
| **Attrition bias -** incomplete outcome data |  | Low | Low | Low | Unclear | Unclear |  |
| **Reporting bias -** selective reporting |  | Low | Low | Unclear | Unclear | Unclear |  |
| **Other bias** |  | Low | Low | Unclear | Unclear | Unclear |  |
| **Overall Risk of Bias** |  | **Low risk** | **Low risk** | **Unclear risk** | **High risk** | **High risk** |  |
| **Newcastle-Ottawa Scale (NOS)** | | | | | | | |
| Selection^1^ | 3 |  |  |  |  |  |  |
| Comparability^2^ | 1 |  |  |  |  |  |  |
| Outcome^3^ | 2 |  |  |  |  |  |  |
| **Total (out of maximum 9)** | **6** |  |  |  |  |  |  |
| ^1^Maximum of 4 stars for: representativeness of the exposed cohort; selection of the non-exposed cohort; ascertainment of exposure; and demonstration that outcome of interest was not present at start of study  ^2^ Maximum of 2 stars for: comparability of cohorts on the basis of the design or analysis  ^3^ Maximum of 3 stars for: assessment of outcome; was follow-up long enough for outcome to occur; and adequacy of follow-up of cohorts | | | | | | | |

**S2 Table. Quality Assessments of Included Studies - continued**

| **Smyth Adapted AE Tool** | | | | | | | |
| --- | --- | --- | --- | --- | --- | --- | --- |
|  | **Malone**  **2005 (34)** | **Milgrom**  **2011 (35)** | **Noonan**  **2009 (36)** | **Pauwels**  **2003 (37)** | **Roux**  **2003 (38)** | **Silverman**  **2006 (39)** | **Skoner**  **2005 (40)** |
| **Study Design** |  |  |  |  |  |  |  |
| Clear study design | Yes | Yes | Yes | Yes | Yes | Yes | NR |
| **Methods to identify AE** |  |  |  |  |  |  |  |
| Detailed methods to identify AE | Yes | NR | Yes | Yes | NR | Yes | NR |
| Detailed data collection methods | NR | NR | Yes | Yes | NR | Yes | NR |
| Clear description of individuals identifying AE | Yes | NR | NR | Yes | NR | Yes | NR |
| **Methods to identify causality** |  |  |  |  |  |  |  |
| Clear description of process to establish causality | NR | NR | NR | NR | NR | Yes | NR |
| Standardized methods used to assess causality | Unclear | NR | NR | NR | NR | NR | NR |
| **Methods to determine preventability** |  |  |  |  |  |  |  |
| Clear description of process used to establish preventability | NR | NR | NR | NR | NR | NR | NR |
| Standardized methods to assess preventability | NR | NR | NR | NR | NR | NR | NR |
| **Methods to determine severity** |  |  |  |  |  |  |  |
| Clear description of process to establish severity | NR | NR | Yes | NR | NR | Unclear | NR |
| Standardized methods to assess severity | Unclear | NR | Unclear | NR | NR | NR | NR |
| **Methods to determine type of AE** |  |  |  |  |  |  |  |
| Clear description of process to establish type of AE | NR | NR | NR | NR | NR | NR | NR |
| Standardized methods to assess type of AE | NR | NR | NR | NR | NR | Unclear | NR |
| **Cochrane Risk of Bias** | | | | | | | |
| **Selection bias -** random sequence generation | Unclear | Low | Unclear | Low | Low | Low | Unclear |
| **Selection bias** - allocation concealment | Unclear | Low | Unclear | Unclear | Low | Unclear | High |
| **Performance bias -** blinding of participants and researchers | Low | Unclear | High | High | High | Low | Unclear |
| **Detection bias -** blinding of outcome assessment | Unclear | Unclear | High | High | Unclear | Low | High |
| **Attrition bias -** incomplete outcome data | Unclear | Low | Low | Low | Low | Low | Unclear |
| **Reporting bias -** selective reporting | Low | Low | Unclear | Low | Low | Low | High |
| **Other bias** | Unclear | Unclear | Unclear | High | Unclear | Low | Unclear |
| **Overall Risk of Bias** | **Unclear risk** | **Low risk** | **High risk** | **High risk** | **High risk** | **Low risk** | **High risk** |
| **Newcastle-Ottawa Scale (NOS)** | | | | | | | |
| Selection^1^ |  |  |  |  |  |  |  |
| Comparability^2^ |  |  |  |  |  |  |  |
| Outcome^3^ |  |  |  |  |  |  |  |
| **Total (out of maximum 9)** |  |  |  |  |  |  |  |
| ^1^Maximum of 4 stars for: representativeness of the exposed cohort; selection of the non-exposed cohort; ascertainment of exposure; and demonstration that outcome of interest was not present at start of study  ^2^ Maximum of 2 stars for: comparability of cohorts on the basis of the design or analysis  ^3^ Maximum of 3 stars for: assessment of outcome; was follow-up long enough for outcome to occur; and adequacy of follow-up of cohorts | | | | | | | |

**S2 Table. Quality Assessments of Included Studies - continued**

| **Smyth Adapted AE Tool** | | | | | | | |
| --- | --- | --- | --- | --- | --- | --- | --- |
|  | **Skoner**  **2008 (41)** | **Skoner**  **2010 (42)** | **vanAdelsberg 2005 (43)** | **Watson**  **1994 (44)** | **Weinstein**  **1997 (45)** | **Wolthers**  **2011 (46)** | **Zarkovic**  **2000 (47)** |
| **Study Design** |  |  |  |  |  |  |  |
| Clear study design | Yes | Yes | Yes | Unclear | Yes | Yes | Yes |
| **Methods to identify AE** |  |  |  |  |  |  |  |
| Detailed methods to identify AE | NR | Yes | Yes | Unclear | Yes | NR | Yes |
| Detailed data collection methods | NR | Yes | NR | Unclear | Yes | NR | Yes |
| Clear description of individuals identifying AE | NR | NR | NR | NR | NR | NR | NR |
| **Methods to identify causality** |  |  |  |  |  |  |  |
| Clear description of process to establish causality | NR | Yes | NR | NR | NR | NR | NR |
| Standardized methods used to assess causality | NR | NR | NR | NR | NR | NR | NR |
| **Methods to determine preventability** |  |  |  |  |  |  |  |
| Clear description of process used to establish preventability | NR | NR | NR | NR | NR | NR | NR |
| Standardized methods to assess preventability | NR | NR | NR | NR | NR | NR | NR |
| **Methods to determine severity** |  |  |  |  |  |  |  |
| Clear description of process to establish severity | NR | Yes | NR | NR | NR | NR | NR |
| Standardized methods to assess severity | NR | NR | NR | NR | NR | NR | NR |
| **Methods to determine type of AE** |  |  |  |  |  |  |  |
| Clear description of process to establish type of AE | NR | NR | NR | NR | NR | NR | NR |
| Standardized methods to assess type of AE | NR | NR | NR | NR | NR | NR | NR |
| **Cochrane Risk of Bias** | | | | | | | |
| **Selection bias -** random sequence generation | Low | Low | Low | Unclear | Unclear | Low | Unclear |
| **Selection bias** - allocation concealment | Low | Unclear | Unclear | Unclear | Unclear | Low | Unclear |
| **Performance bias -** blinding of participants and researchers | Low | Low | Low | High | Low | High | High |
| **Detection bias -** blinding of outcome assessment | Low | Unclear | Low | High | Unclear | Low | High |
| **Attrition bias -** incomplete outcome data | Low | Unclear | Low | Unclear | Low | Low | Low |
| **Reporting bias -** selective reporting | Low | Low | Low | Low | Low | Low | Low |
| **Other bias** | Low | Low | Low | High | Unclear | Unclear | Low |
| **Overall Risk of Bias** | **Low risk** | **Low risk** | **Low risk** | **High risk** | **Unclear risk** | **High risk** | **High risk** |
| **Newcastle-Ottawa Scale (NOS)** | | | | | | | |
| Selection^1^ |  |  |  |  |  |  |  |
| Comparability^2^ |  |  |  |  |  |  |  |
| Outcome^3^ |  |  |  |  |  |  |  |
| **Total (out of maximum 9)** |  |  |  |  |  |  |  |
| ^1^Maximum of 4 stars for: representativeness of the exposed cohort; selection of the non-exposed cohort; ascertainment of exposure; and demonstration that outcome of interest was not present at start of study  ^2^ Maximum of 2 stars for: comparability of cohorts on the basis of the design or analysis  ^3^ Maximum of 3 stars for: assessment of outcome; was follow-up long enough for outcome to occur; and adequacy of follow-up of cohorts | | | | | | | |

**S2 Table. Quality Assessments of Included Studies - continued**

| **Smyth Adapted AE Tool** | | | | | | | |
| --- | --- | --- | --- | --- | --- | --- | --- |
|  | **Abusamra**  **2013 (48)** | **Baumann**  **2014 (49)** | **Cavkaytar**  **2015 (50)** | **Egeland**  **2013 (51)** | **Erdem**  **2015 (52)** | **Fagbuyi**  **2016 (53)** | **Kenyon**  **2013 (54)** |
| **Study Design** |  |  |  |  |  |  |  |
| Clear study design | NR | Unclear | Yes | Yes | Yes | Yes | Yes |
| **Methods to identify AE** |  |  |  |  |  |  |  |
| Detailed methods to identify AE | Yes | Yes | Yes | Yes | NR | Yes | NR |
| Detailed data collection methods | NR | NR | Yes | NR | NR | Yes | Yes |
| Clear description of individuals identifying AE | NR | NR | NR | NR | NR | Yes | NR |
| **Methods to identify causality** |  |  |  |  |  |  |  |
| Clear description of process to establish causality | NR | NR | NR | Yes | NR | NR | NR |
| Standardized methods used to assess causality | NR | NR | NR | NR | NR | NR | NR |
| **Methods to determine preventability** |  |  |  |  |  |  |  |
| Clear description of process used to establish preventability | NR | NR | NR | NR | NR | NR | NR |
| Standardized methods to assess preventability | NR | NR | NR | NR | NR | NR | NR |
| **Methods to determine severity** |  |  |  |  |  |  |  |
| Clear description of process to establish severity | NR | NR | NR | NR | NR | NR | NR |
| Standardized methods to assess severity | NR | NR | NR | NR | NR | NR | NR |
| **Methods to determine type of AE** |  |  |  |  |  |  |  |
| Clear description of process to establish type of AE | NR | NR | NR | NR | NR | Yes | Yes |
| Standardized methods to assess type of AE | NR | NR | NR | NR | NR | NR | NR |
| **Cochrane Risk of Bias** | | | | | | | |
| **Selection bias -** random sequence generation | (Abstract) | (Abstract) |  |  |  |  |  |
| **Selection bias** - allocation concealment |  |  |  |  |  |  |  |
| **Performance bias -** blinding of participants and researchers |  |  |  |  |  |  |  |
| **Detection bias -** blinding of outcome assessment |  |  |  |  |  |  |  |
| **Attrition bias -** incomplete outcome data |  |  |  |  |  |  |  |
| **Reporting bias -** selective reporting |  |  |  |  |  |  |  |
| **Other bias** |  |  |  |  |  |  |  |
| **Overall Risk of Bias** |  |  |  |  |  |  |  |
| **Newcastle-Ottawa Scale (NOS)** | | | | | | | |
| Selection^1^ | (Abstract) | (Abstract) | 4 | 4 | 2 | 2 | 4 |
| Comparability^2^ |  |  | 2 | 1 | 0 | 0 | 0 |
| Outcome^3^ |  |  | 4 | 3 | 0 | 3 | 3 |
| **Total (out of maximum 9)** |  |  | **10** | **8** | **2** | **5** | **7** |
| ^1^Maximum of 4 stars for: representativeness of the exposed cohort; selection of the non-exposed cohort; ascertainment of exposure; and demonstration that outcome of interest was not present at start of study  ^2^ Maximum of 2 stars for: comparability of cohorts on the basis of the design or analysis  ^3^ Maximum of 3 stars for: assessment of outcome; was follow-up long enough for outcome to occur; and adequacy of follow-up of cohorts | | | | | | | |

**S2 Table. Quality Assessments of Included Studies - continued**

| **Smyth Adapted AE Tool** | | | | |
| --- | --- | --- | --- | --- |
|  | **Perry**  **2014 (55)** | **Sarniak**  **2013 (56)** | **Stempel**  **2016 (57)** | **Wisecup**  **2015 (58)** |
| **Study Design** |  |  |  |  |
| Clear study design | NR | Yes | Yes | Yes |
| **Methods to identify AE** |  |  |  |  |
| Detailed methods to identify AE | Yes | Yes | Yes | Yes |
| Detailed data collection methods | NR | Yes | Yes | Yes |
| Clear description of individuals identifying AE | NR | NR | Yes | NR |
| **Methods to identify causality** |  |  |  |  |
| Clear description of process to establish causality | NR | NR | NR | Yes |
| Standardized methods used to assess causality | NR | NR | NR | NR |
| **Methods to determine preventability** |  |  |  |  |
| Clear description of process used to establish preventability | NR | NR | NR | NR |
| Standardized methods to assess preventability | NR | NR | NR | NR |
| **Methods to determine severity** |  |  |  |  |
| Clear description of process to establish severity | Yes | NR | Yes | NR |
| Standardized methods to assess severity | NR | NR | NR | NR |
| **Methods to determine type of AE** |  |  |  |  |
| Clear description of process to establish type of AE | NR | NR | NR | NR |
| Standardized methods to assess type of AE | NR | NR | NR | NR |
| **Cochrane Risk of Bias** | | | | |
| **Selection bias -** random sequence generation | (Abstract) |  | Low |  |
| **Selection bias** - allocation concealment |  |  | Low |  |
| **Performance bias -** blinding of participants and researchers |  |  | Low |  |
| **Detection bias -** blinding of outcome assessment |  |  | Low |  |
| **Attrition bias -** incomplete outcome data |  |  | Low |  |
| **Reporting bias -** selective reporting |  |  | Low |  |
| **Other bias** |  |  | Low |  |
| **Overall Risk of Bias** |  |  | **Low Risk** |  |
| **Newcastle-Ottawa Scale (NOS)** | | | | |
| Selection^1^ | (Abstract) | 2 |  | 4 |
| Comparability^2^ |  | 0 |  | 0 |
| Outcome^3^ |  | 2 |  | 2 |
| **Total (out of maximum 9)** |  | **4** |  | **6** |
| ^1^Maximum of 4 stars for: representativeness of the exposed cohort; selection of the non-exposed cohort; ascertainment of exposure; and demonstration that outcome of interest was not present at start of study  ^2^ Maximum of 2 stars for: comparability of cohorts on the basis of the design or analysis  ^3^ Maximum of 3 stars for: assessment of outcome; was follow-up long enough for outcome to occur; and adequacy of follow-up of cohorts | | | | |
